# Reliability of toxicokinetic modelling for PFAS exposure assessment in contaminated water in northern Italy

**DOI:** 10.1016/j.heliyon.2024.e35288

**Published:** 2024-07-31

**Authors:** L. Vaccari, A. Ranzi, C. Canova, G. Ghermandi, S. Giannini, G. Pitter, F. Russo, J. Stefanelli, S. Teggi, A. Vantini, M.Z. Jeddi, T. Fletcher, A. Colacci

**Affiliations:** a Center for Environment, Prevention and Health, Emilia-Romagna Regional Agency for Prevention, Environment and Energy (Arpae), 40139, Bologna, Italy; b Unit of Biostatistics, Epidemiology and Public Health-University of Padua, Padua, Italy; c Department of Engineering “Enzo Ferrari”, University of Modena and Reggio Emilia, 41125, Modena, Italy; d Screening and Health Impact Assessment Unit, Azienda Zero, Veneto Region, Padua, Italy; e Directorate of Prevention, Food Safety and Veterinary Public Health, Veneto Region, Venice, Italy; f Agency for Prevention and Protection of the Environment of the Veneto Region (ARPAV), 35121, Padova, Italy; g National Institute for Public Health and the Environment (RIVM), 3720 BA, Bilthoven, the Netherlands; h London School of Hygiene and Tropical Medicine (LSHTM), London, UK

**Keywords:** TK modelling, PBTK modelling, PFOA, PFOS, Exposure assessment, Risk assessment, Water contamination

## Abstract

**Introduction:**

Long-term contamination of tap water and groundwater by perfluoroalkyl and polyfluoroalkyl substances (PFASs) has been documented in the Veneto region of northern Italy. This study aimed to assess the exposure of individuals residing in the contaminated area and to test several toxicokinetic (TK) models of varying complexities to identify an efficient method for predicting perfluorooctanoic acid (PFOA) and perfluorooctanesulfonic acid (PFOS) concentrations in human serum using observed data.

The ultimate goal is to provide public health officials with guidance on selecting the appropriate TK model for specific contexts, a reliable and rapid tool to support human bio-monitoring (HBM) studies.

**Methods:**

Two simpler empirical TK models and a more complex multi-compartment physiologically based toxicokinetic (PBTK) model were compared with individual and aggregate data from an HBM study. In addition, the PBPK model was modified by adjusting input parameters and introducing new terms into the equations within the original model code. These modifications aimed to optimize the results compared to the original model, with some versions incorporating adjustments to account for the influence of menstruation in women. All models were evaluated to understand their strengths and weaknesses, providing guidance on the appropriate model to use according to specific scenarios.

**Results:**

The results obtained from the tested models were quite similar, with significant improvements observed only in the modified models. Simpler models also provided satisfactory results in scenarios involving low PFOS serum concentrations and recent exposure cessation. In many cases, predictions demonstrated high accuracy, particularly at the aggregate level and for women.

**Conclusions:**

These findings suggest that environmental protection agencies and health authorities may benefit from employing the tested models at the aggregate level as an initial step in HBM studies, rather than conducting more invasive and expensive screening campaigns.

## Highlights

• The exposure to PFOA and PFOS in drinking water and food was investigated.
• Two empirical TK models and one PBTK model were tested on HBM data and evaluated.
• A multi-compartment PBTK model was optimized modifying parameters and equations.
• Solutions were proposed to address exposure assessment and model evaluation issues.
• PFAS were well predicted by the tested models, especially the modified ones.

## Graphical abstract

**Figure:**
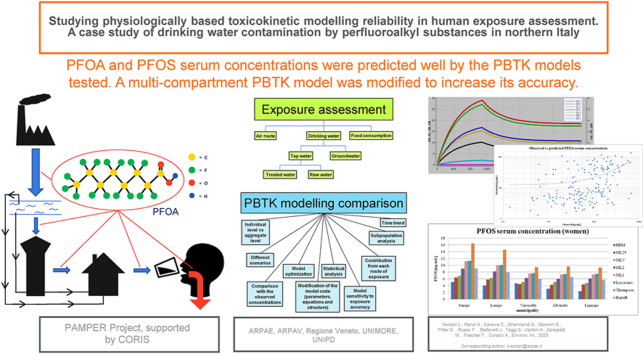

## Abbreviations

BCS: best-case scenario
CA: PFOS concentration in plasma
Free: free fraction of PFOS in plasma
GAC: Granular Activated Carbon
HBM: human bio-monitoring
ML1: modified Loccisano
ML2: modified Loccisano
ML29: modified Loccisano
MLS: most-likely scenario
MLV: modified Loccisano
N: menstruation coefficient
N_fert_: menstruation coefficient for fertile age
N_fert,V_: menstruation coefficient for fertile age derived from Verner et al. [53]
QCP: plasma flow in humans
PBTK: physiologically based toxicokinetic
PFASs: perfluoroalkyl and polyfluoroalkyl substances
PFOA: perfluorooctanoic acid
PFOS: perfluorooctanesulfonic acid
TK: toxicokinetic
WCS: worst-case scenario
WRS: Wilcoxon rank-sum

## 1 Introduction

Perfluoroalkyl and polyfluoroalkyl substances (PFASs) are a group of over 9000 man-made chemicals characterized by fully or partly fluorinated carbon chains bonded to various functional groups [1]. Due to their chemical and thermal stability, as well as water and oil resistance, PFASs have been widely used in numerous industrial processes. These applications include non-stick cookware, furniture, household cleaners, clothing, fire-fighting foam, ammunition, climbing ropes, guitar strings and many other common products [2]. Furthermore, the strong carbon-fluoride bonds in PFASs confer resistance to metabolic transformation and environmental degradation [3].

For these reasons, PFASs have been detected in all environmental matrices globally [[4], [5], [6], [7], [8], [9], [10], [11]]. A significant mode of human exposure is through the ingestion of contaminated drinking water, with their presence reported in tap water worldwide [12].

In the Veneto region of northern Italy, widespread contamination of drinking water by PFASs has been an ongoing issue, likely starting in the late 1960s. High concentrations of PFASs remain in rivers and groundwater, with over 130,000 individuals chronically exposed [13,14]. PFASs are classified as emerging contaminants [15]and pose health risks to humans. Numerous epidemiological studies have suggested associations between PFASs exposure and increased serum lipids (e.g. total cholesterol), elevated serum hepatic enzymes, reduced serum bilirubin levels, decreased antibody response, and pregnancy-induced hypertension in adults [4,16]. In children, exposure is associated with cardiometabolic, thyroid and renal diseases, neurodevelopmental disorders, attention deficit disorder, infections, puberty onset, dyslipidemia, immunity issues (including vaccine response and asthma), renal function impairment, and altered age at menarche in children [17]and with low birth weight in newborns [4]. Furthermore, increases in testicular and kidney cancer have been observed in highly exposed humans [4,18] and certain PFASs are considered possibly carcinogenic to humans [[19], [20], [21]].

Despite significant interest in PFASs, the epidemiology and toxicology of these chemicals are still limited and their toxicokinetics and underlying mechanisms are poorly understood [22,23]. PFASs’ persistence in the environment suggests bioaccumulation in the liver of living organisms [24], including humans. For compounds like perfluorooctanoic acid (PFOA) and perfluorooctanesulfonic acid (PFOS), absorption, distribution, metabolism and excretion processes seem to be supported by organic anion transporters, aiding gastrointestinal absorption, tissue uptake, and renal excretion and reabsorption [4,[25], [26], [27]]. Understanding these processes, as well as identifying main exposure routes, is crucial for assessing real human risk and establishing scientific-based approaches for risk management. Moreover, developing an accurate and reliable health risk assessment framework to investigate population risk from environmental pollutants is a key issue in contemporary risk assessment science [28]. In addition, using a fast method to estimate risk for a target population is vital for local authorities and environmental protection agencies to design and implement timely, efficient and cost-effective health interventions and site remediation.

Toxicokinetic (TK) models are valuable tools for this purpose [29]. These mathematical models, based on empirical or toxicology-based equations, predict the effective dose in organs and tissues from contaminant intake into the human body [30]. They provide a swift and reasonably accurate assessment of contamination impact on exposed populations. Many studies have investigated the relationship especially between PFOA and PFOS (PFAS) concentrations in water and their level in human serum using TK models [[31], [32], [33]].

There are various types of PFAS-specific TK models -i.e. models specifically developed to simulate PFAS- with differing levels of complexity [29]. However, the differences in reliability and accuracy between more complex and simpler models are still not well understood.

Identifying which models are most suitable for specific scenarios, considering model complexity and the time and resources required to obtain predictions, is crucial for public health professionals, particularly those without specialized expertise in TK models. Indeed, these models can help achieve the goal of obtaining sufficiently accurate PFAS serum concentration estimates as quickly and efficiently as possible.

Therefore, our research focused on developing an effective strategy for exposure assessment in cases of widespread PFAS contamination and testing TK models of varying complexity to identify an efficient method for predicting PFAS concentrations in human serum.

The principal aim of our research was to provide insights into the advantages and disadvantages of using PFAS-specific TK models of varying complexity for contamination scenarios similar to those described in this study.

In addition to the accuracy and precision of the predicted results, various parameters were considered to understand the strengths and weaknesses of the tested models, providing guidance on the most cost-effective model to use: model complexity (i.e. level of expertise necessary to set up the software parameters and run simulations), model reliability, type and amount of input data required (i.e. difficulty in collecting accurate exposure assessment data), time required to set up simulations, and economic cost to obtain data.

The development of a reliable and efficient procedure for assessing environmental exposure and internal PFAS doses ultimately aims to support human bio-monitoring (HBM) programs for public health, potentially replacing expensive, invasive and time-consuming HBM screening campaigns in the near future.

Various analyses were conducted to compare model results, assess the importance of certain exposure variables, and evaluate the uncertainty associated with predicted values. Additionally, we examined the influence of sex on the toxicokinetic profile by comparing PFAS concentrations in men and women to determine the potential role of menstruation in PFAS excretion pathways.

The research presented in this article is part of the “Pharmacokinetics Modelling for PFAS Exposure and related Risk” (PAMPER) project.

This project aims to test and optimize several PFAS-specific TK models and highlight the toxicological behavior and health risks associated with PFAS exposure. The project's overarching goal is to create a comprehensive system for collecting and processing information to fully understand the fate of PFAS in contaminated areas, thereby supporting regional public health policies in the Veneto region. Additionally, the project framework is intended to be exportable to other situations and areas, applicable to other actual or presumed environmental contaminations. Integrating data on PFAS toxicological profiles, effective concentrations, toxicokinetics, and fate in the human body will help define environmental concentration limits and provide useful indications of potential damage time windows in humans from acute and chronic exposures.

The project encompassed two main components. One focusing on population exposure assessment and the use, validation and modification of several TK models, is the subject of our research described in this paper. The other component involved in vitro assessment of carcinogenic potential of PFAS to inform toxicodynamic pathways [34].

## 2 Methods

### 2.1 Study population

**Fig. 1:**
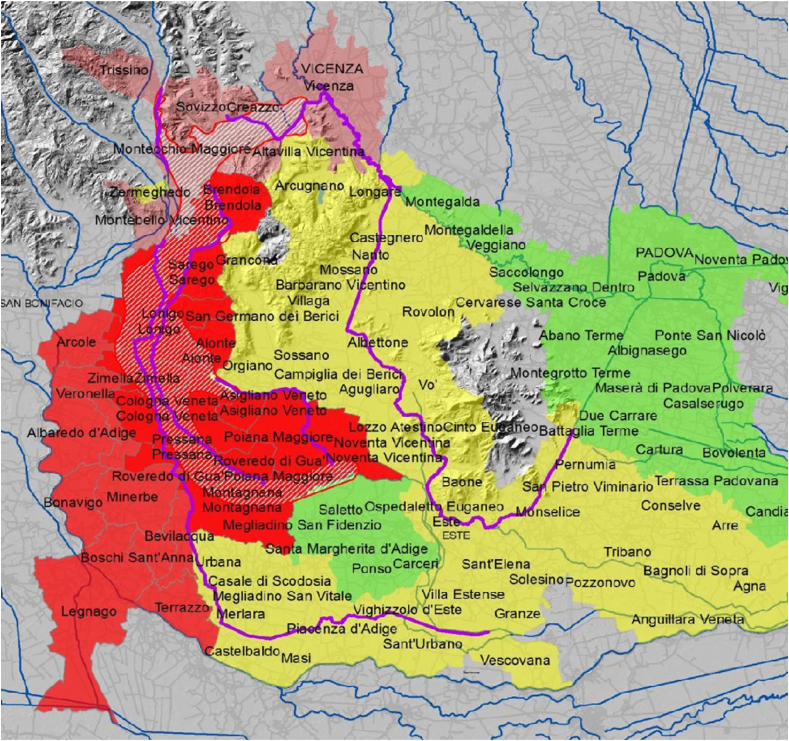
Areas of different exposure and different risk for health. Dashed area = contamination plume in the groundwater, red area = area of maximum exposure (divided in the A-red area: dark red, and the B-red area: light red), orange area = independent water harvesting, yellow area = warning area, green area = in-depth area (image from Refs. [13,14]).

For this study, the Veneto Region and University of Padua provided us with the human data in an anonymous format. These data were collected from the participants enrolled in a HBM study [35]. 179 individuals aged 14–39 years (mean: 26) were selected from among these subjects. Selection criteria were established to assess the reliability of TK models under various conditions. Therefore, an equal number of males (88) and non-pregnant females (91) of childbearing age was chosen to ensure comparability. In addition, subjects were selected from five municipalities: Sarego (25), Lonigo (51), Legnago (71), Veronella (15), and Albaredo d’Adige (17), to reflect diverse exposure scenarios and thus the value and uncertainty associated with the parameter inputs into the models (section 2.2.1.1). Sarego and Lonigo were located in a relatively high-exposure area, known as the “A-red area” (Fig. 1), where both tap water and groundwater were contaminated with PFAS, while the other municipalities were in the “B-red area” (Fig. 1), with only tap water contamination. Median PFAS serum levels for residents varied between the five municipalities, enabling model testing across a broad range of values [13,14,36]. Additionally, to ensure comparability between municipalities, the analysis was stratified by sex and blood sampling dates were within a narrow timeframe (averaging no more than an 8-month difference between municipalities) and considered in the analysis of the TK models' output.

### 2.2 Exposure assessment

In North America and Europe, teenagers and adults predominantly experience chronic PFASs exposure through dietary intake, including drinking water [7,37,38], which appears to be the primary source of PFASs in contaminated communities [24]. Conversely, dermal contact seems to be a negligible route of exposure [39]. Therefore, the dermal route was not investigated in this study.

The air route can be a significant exposure pathway for PFASs [15,[40], [41], [42]]. However, in this study the primary medium for long-range environmental transport was drinking water, while the air route was deemed negligible for the subjects living in the contaminated area after a modelling simulation of PFOA dispersion in the atmosphere [13,14].

#### 2.2.1 PFAS intake through water

Monthly PFAS concentration data for groundwater, raw water (not passing through the Granular Activated Carbon (GAC) filters) and treated water (after passing through the GAC filters in the water treatment plant) were collected [13]. The subjects were potentially exposed to PFAS from groundwater through a private well and to PFAS in tap water first through the raw water (before the GAC filters installation in 2013) and subsequently through the treated water [13,14].

The PFAS concentration trend observed in tap water (Annex A) was shown by grouping the data into several different time periods and calculating a representative value for each of them (corresponding to the average of the concentration data collected in that period). The process aimed to find a quick and efficient way to provide accurate input data to the multi-compartment model. In this way, the significant decrease in the PFAS concentration observed in tap water during the last years of exposure was described in the PBTK model as the variation of the representative value of PFAS concentration through the different periods.

The first selected period referred to the time frame in which the PFAS concentration reached the highest values and it was assumed to start before the beginning of the exposure for all the subjects involved and to end in correspondence with the first significant and extended decrease in concentration observed in tap water (due to the effect of the GAC filters installation and/or other remediation actions). The representative value for this period was chosen as the average of the PFAS concentrations observed in the first few months after the detection of PFAS in groundwater. In fact, the concentrations observed in that time frame were considered also representative of the ongoing exposure before July 2013, when no PFAS measurements were available. This choice was supported by the analysis of the groundwater flux and the history of PFAS discharge from a chemical plant [13,14,36], and it was confirmed by the slow downward trend of the PFAS concentration in the raw water (Annex A). The results of the analysis indicated that steady concentrations levels have likely existed in the study area for several decades, since the contamination was traced back to the 1960s.

All the observed values below the limit of detection (LOD) for PFAS in tap water and raw water (5 or 10 ng/L) were assumed to be equal to the LOD, to offset the underestimation due to the shortness of the time series.

The starting and final dates for the other periods were chosen on the basis of the evident variations in the PFAS concentrations. (Tab. A.6, Annex A).

The concentrations of PFOA detected in groundwater exhibited considerable variation, with higher levels observed in the A-red area and significantly lower levels in the B-red area, compared to those in tap water in both red areas. The temporal trend of PFAS concentrations in groundwater was considered non-significant due to substantial spatial variability in all three dimensions of the sampling locations. The representative PFOA concentration in groundwater for the A-red area was chosen as the median of the observed distribution, as most data points fell below the mean. Moreover, all the values below the LOD (5 or 10 ng/L) were assumed to be equal to the LOD, potentially leading to an overestimation. However, the average value was also considered in the simulation step due to the unknown exact location of the subjects within the municipality. For Legnago, the representative PFOA concentration in groundwater was the average, while for Albaredo and Veronella it was assumed to be 0 ng/L (Tab. A.9, Annex A).

The exposure to PFOS in groundwater for the subjects living in the B-red area was assumed to persist until the date of blood sampling to compensate for potential underestimation resulting from adopting a concentration value below the LOD. The average of the observed data was selected as the representative value for PFOS concentrations in groundwater in the A-red area (Tab. A.10, Annex A).

##### 2.2.1.1 Exposure scenarios

The exposure assessment encountered some significant challenges (section 3.7).

The limited knowledge of the drinking habits of the subjects living in the A-red area was concern, due to the different PFOA concentrations in bottled water, tap water and groundwater. The same problem, to a much lesser extent, also affected those residing in Legnago. In fact, that municipality was classified in the B-red area [13]; however, analysis of groundwater showed an average PFOA concentration slightly exceeding the LOD of 19 ng/L.

The issue was addressed with the creation of three different exposure scenarios for Sarego, Lonigo and Legnago: the Worst-case scenario (WCS), the Most likely scenario (MLS) and the Best-case scenario (BCS). (Table 1).

**Table 1:** Characteristics of the exposure scenarios. WCS = Worst-case scenario, MLS= Most likely scenario, BCS= Best-case scenario. WI = water intake, TW = tap water, GW = groundwater.

Scenario	Fraction of WI			End of exposure to private well	Change in water use
	From private well	From TW	From bottled water		
WCS	1/3	1/3	1/3	at the time of the sample collection	NO
MLS	1/3	1/3	1/3	at the end of the first time period	After the detection of PFAS in GW (2013)
BCS	–	1/3	2/3	–	NO

After the analysis of the concentration data (Tab. A.9, Annex A), for the subjects in the A-red area, both the median and the average concentrations were considered meaningful representative values for PFOA in groundwater. Therefore, they were both used in the analyses.

These scenarios aimed to find an uncertainty range associated with the concentrations predicted by the TK models related to water intake (sections 3.5.2, 3.7, Tab. A.14, Annex A).

The daily intake of PFAS through drinking water was computed by multiplying the sum of the PFAS concentration values in tap water and groundwater by the corresponding water intake rates (Annex A).

#### 2.2.2 PFAS intake through food

Data on the number of food portions taken weekly were collected in the HBM questionnaire. The “standard portion” size for each type of food was taken from the Italian Society of Human Nutrition that defines the quantitative standards for portions in Italy in accordance with consumer expectations. Then, the number of portions of food per week taken for each subject was multiplied by the weight of the “standard portion” for each type of food to obtain the weight of food ingested weekly. (Tab. A.15, A.16, A.17; Annex A).

PFAS concentrations in food were taken from a study [43] on the Veneto population exposed to PFAS contamination. The concentration values referred to local food since people lived in a rural area and many subjects owned vegetable gardens (at least 71 subjects) and animals (at least 26 subjects). In the study many samples from different types of food were analyzed for each food category and three scenarios were developed. The chosen PFAS concentration value for each food category was the average of the data in the medium scenario.

### 2.3 TK models

In this study a PFAS-specific multi-compartment model [44], supported by detailed data from the exposure assessment, and two simpler PFAS-specific empirical one-compartment models (the “Thompson model” [45] and the “Bartell model” [46]), were tested using individual and aggregate-level analyses.

The predicted PFAS serum concentrations were compared with data observed in the HBM study [35] to evaluate the models' accuracy. Furthermore, the code of the multi-compartment model was modified by adjusting the input parameters and adding specific terms to the system of differential equations, resulting in modified models that improved accuracy -i.e., reduced deviation from the observed data- (Table 3 and Results).

**Table 2:** Values of parameters associated with the menstrual cycle calculated from the corrected values of the menstruation coefficient taking into account the fertile age (N_fert_) calculated for women in each municipality. Q_b,mc,fert_ = average blood volume lost with menstruation during one cycle, corrected for fertile age; Q_p,year,fert_ = average plasma volume lost with menstruation during one year, corrected for fertile age, considering a number of 12.5 cycles per year; Q_p,mc,fert_ = average plasma volume lost with menstruation during one cycle, corrected for fertile age; Q_p,mc_ = average plasma volume lost during a menstrual cycle; Q_p,h_ = average plasma volume hourly lost with menstruation. age_fert_ = fertile age in women until blood sampling (years), which was calculated as the difference between the average age of women in that municipality and the beginning of the fertile age (12, according to Ref. [47]). Age = average age for the women living in that municipality. Exp_t_ = time of exposure for women living in that municipality.

Municipality	Q_b,mc,fert_ (mL/cycle)	Q_p,year,fert_ (mL/year)	Q_p,mc,fert_ (mL/cycle)	Q_p,mc_ (mL/cycle)	Q_p,h_ (mL/h)	Age_fert_ (years)	Age (years)	Exp_t_ (years)
Sarego	59.4	1158	92.7	74.9	1.07E-04	14.8	26.8	18.3
Lonigo	60.9	1188	95.0	85.3	1.22E-04	15.0	27.0	16.7
Veronella	79.0	1540	123.2	87.9	1.25E-04	14.2	26.2	19.9
Albaredo	107.9	2105	168.4	90.9	1.30E-04	9.5	21.5	17.6
Legnago	70.4	1372	109.8	99.8	1.42E-04	16.0	28.0	17.6
Women (total population)	75.7	1476.5	118.1	88.0	1.28E-04	14.9	26.9	17.7

**Table 3:** Versions of the Loccisano model created in the present work and modifications carried out from the previous version. Models: ML29 = Modified Loccisano 29 %, MLV = Modified Loccisano-Verner, ML2 = Modified Loccisano Version 2, ML1 = Modified Loccisano Version 1. Free = free fraction of PFOS in plasma, Kt = resorption affinity constant, Tmc = maximum resorption rate (Tmc).

Model name	initial model	Section in which the modifications were carried out	Modifications from the previous version of the model
Adapted Loccisano	Original Loccisano	exposure parameters exposure periods oral exposure dose equation for gut compartment method to solve the system of differential equations physiokinetic parameters	introduction of different exposure periods, description of the beginning and the end of the exposure, duration of dose, addition of terms in the equation for gut compartment to describe the differences in PFAS intake through time periods, adoption of the Stiff method, cardiac outputs, T_mc_
ML1	Adapted Loccisano	physiokinetic parameters	human-based partition coefficients
ML2	ML1	physiokinetic parameters	T_mc_, Free
ML29	ML2	equation for plasma compartment	Addition of menstruation term
MLV	ML29	equation for plasma compartment	Modification of the menstruation parameter in the menstruation term

The code of the multi-compartment model for humans developed by Loccisano et al. [44], named the “original Loccisano model”, was first modified (Table 3 and Annex B) to describe the rapid change in PFAS intake during the last years before the end of exposure due to the decrease in PFAS concentrations in tap water.

Subsequently, this new version of the Loccisano model, named the “Adapted Loccisano model”, was modified by changing partition coefficients -“modified Loccisano model Version 1” (ML1)- and by adjusting several input parameters -“modified Loccisano model Version 2” (ML2)- to reduce the deviation of the predicted values from the observed data (section 3.8). The “calibration” of the model (i.e. a sort of optimization using the HBM data) involved updating certain parameters with more recent values found in the literature [44,[48], [49], [50], [51], [52], [53], [47], [54]] to ensure their “physiological validity”. (Table 3).

Starting from the ML2 model, an equation of the model code was modified to account for the lower PFAS serum concentrations in women due to menstruation (section 3.3 and Table 3).

Finally, the parameters describing the renal process -i.e. resorption affinity constant (K_t_) and maximum resorption rate (T_mc_)- were optimized in ML2-based models (section 3.4).

All the simulations were run using the Berkeley-Madonna (B-M) program language [55].

The evaluation of the model performance was based on the comparison of the PFAS serum concentrations observed at the time of the blood sampling with the predicted PFAS concentrations in the plasma compartment, for the Loccisano-based models, or with the predicted serum concentrations, for the one-compartment models, since comparing PFAS concentrations in plasma and in serum is equivalent [56].

The models tested in this study were chosen after an in-depth analysis of the literature on PBTK models implemented specifically for PFASs. The analysis aimed at highlighting the model advantages and deficits and at collecting values for several parameters required as input in the models to use them in the calibration process. The analysis of the literature (Annex A) was performed by searching on Web of science, Scopus and PubMed for studies on published PBTK models for PFAS in humans.

A sensitivity analysis was performed for the original Loccisano model [57] and other Loccisano-based models by Refs. [51,52]. Given the adjustments made in the modified models described in this study, a new sensitivity analysis was deemed unnecessary. However, the impact of the parameters describing the renal resorption process (K_t_ and T_mc_) on the final result was evaluated during the optimization phase.

#### 2.3.1 Analysis

The models were compared by apply them in turn to predict PFAS serum concentrations.

The analyses provided a comparison of the models according to several exposure scenarios created to determine the level of uncertainty associated with some parameters in the final result. Starting from the scenarios created in the exposure assessment (WCS, MLS, BCS), combined scenarios were designed to take into account all the possible situations (WCS + AS, MLS + AS, BCS + AS, MLS + MS, where AS = average scenario and MS = median scenario). The combined scenarios differed in terms of the value chosen to represent the concentration of PFOA in groundwater (i.e. the average or the median) and the differences in the time of exposure to PFOA in groundwater. Therefore, these scenarios varied only for the subjects residing in the A-red area and in Legnago. The MLS + MS was assumed to be the most reliable scenario.

The primary analysis involved comparing the predicted and observed PFAS serum concentrations using both individual and aggregate data. In addition, a comparison was conducted between the results obtained from individual-level versus aggregate-level analysis to determine the most appropriate approach. Another key analysis focused on comparing combined scenarios to understand the range of uncertainty for PFOA serum concentration predictions. Furthermore, a subset of the total population was examined to ensure the reliability of the findings. Additionally, the predicted trend of PFAS serum concentrations over time was investigated to assess values before and after the blood sampling period. Moreover, the contribution of each route of exposure to the predicted serum concentration was estimated using the model with the best performance. Last, PFOA concentrations in various tissues of the human body were predicted in anticipation of collecting observed data for other organs in the near future.

## 3 Results and discussion

### 3.1 Population analysis

The observed serum concentrations in the enrolled subjects were very high for PFOA (10th percentile-median-average-90th percentile: 6.3-39.1-57.9–124.9 ng/mL) and quite low for PFOS (10th percentile-median-average-90th percentile: 1.6-3.3-4.4–8.7 ng/mL) with respect to the Italian general population (median-90th percentile: 3.59–6.92 ng/g for PFOA and 6.31–12.38 ng/g for PFOS [58]) and considerable variability was detected for PFOA (st. dev. 62.3 ng/mL, while 3.4 ng/mL for PFOS).

Sex was considered in each comparative analysis since serum PFAS concentrations were considerably higher in men (median and av. ± st. dev. (ng/mL): 57.0, 78.0 ± 71.4 in men and 23.2, 38.5 ± 44.4 in women for PFOA; 4.0, 5.2 ± 3.3 in men and 2.6, 3.7 ± 3.4 in women for PFOS) after a WRS test with continuity correction (p-value = 5.598E-07 for PFOA and = 4.017E-06 for PFOS). The choice of using the WRS test was based on the size of the samples and on the result of a Shapiro-Wilk test that showed a non-normality for all the distributions of serum concentration data. The tests were run with R software [59].

Independent sample two-tailed t-tests showed that age and exposure time were not significantly different between men and women (p-value: 0.29 for age and 0.41 for exposure time) while the water intake rate and body weight were greater for men (p-values: 0.29E-04 and 5.9E-15, respectively).

PFAS serum concentrations observed in the subjects in the A-red area were significantly greater than those observed in the subjects living in the B-red area after a WRS test with continuity correction (p-value = 2.46E-05 for PFOA; p-value = 1.04E-03 for PFOS) and independent samples (two-tailed or one-tailed) t-tests did not show a significant difference in the distributions of the data for any of the exposure variables investigated (age: p-value = 0.68, exposure time: p-value = 0.47, water intake: p-value = 0.26 and weight: p-value = 0.10). Therefore, the difference in PFAS serum concentrations was likely not due to a difference in physical characteristics or exposure between the subjects of the two groups, except for PFAS concentrations in water.

Residents of Sarego exhibited the highest serum concentrations of PFAS (median: 73.3 ng/mL for PFOA; 5.7 ng/mL for PFOS), whereas concentrations in the subjects living in Albaredo, Lonigo, and Veronella were notably lower. Conversely, residents of Legnago demonstrated the lowest serum concentrations (median: 20.3 ng/mL for PFOA; 2.7 ng/mL for PFOS). (Tab. A.2, Annex A).

### 3.2 Exposure assessment

For PFOA, the daily average intake through drinking water was greater with respect to PFOS (188.4 vs 16.7 ng/day) and differed consistently between municipalities -ranging from 381.7 (Sarego) to 26.2 (Legnago) ng/day-with slightly lower values for women. In contrast, the daily average total intake through food was quite similar for PFOA and PFOS (43.3 vs 37.2 ng/day), for all municipalities -ranging from 37.1 (Albaredo) to 51.0 (Lonigo) ng/day- and for men and women (42.1 vs 44.5 ng/day respectively, for PFOA). However, for both PFAS, it was slightly greater for the subjects in the A-red area. Therefore, the sex differences observed in the PFAS serum concentrations were not attributed to food, while the difference between the two red areas was attributable to food only to a small extent.

Nevertheless, for PFOS (and PFOA for the municipalities in the B-red area) the contribution of the food route to the total exposure was relevant, since the intake through food was similar to that through water. (Annex A).

The average PFAS total daily intake was much greater for PFOA (231.8 ng/day) than for PFOS (53.9 ng/day) for the subjects in the A-red area than for those in the B-red area – PFOA: 414.9 ng/day (MS) vs 98.0 ng/day, PFOS: 70.8 ng/day vs 41.5 ng/day – and no sex differences were found (Tab. A.18, Annex A).

### 3.3 The modifications to the original Loccisano model

In addition to the modifications made to the original Loccisano model (Table 3 and Annex B), the rat-based partition coefficients proposed by Loccisano et al. [44] were replaced with the human-based coefficients proposed by Fàbrega (et al., 2014) to evaluate potential improvements in the predicted values. (Annex C).

Subsequently, starting from this model –the ML1 (section 2.3)-, the ML2 model was implemented adjusting T_mc_ from 6000 μg/h/kg^0.75^ [60] to 10000 μg/h/kg^0.75^ [61] for PFOA and from 3500 μg/h/kg^0.75^ [44] to 3270 μg/h/kg^0.75^ [48] for PFOS. Moreover, in the ML2 for PFOS, the free fraction of PFOS in plasma (Free) was adjusted from 0.025 to 0.03 [49].

The decision to modify these highly sensitive variables [50,51] was justified by specific reasons (section 3.8 and Annex C).

#### 3.3.1 The modified Loccisano 29 model

A further adjustment of the ML2 model was made based on the lower PFAS serum concentrations observed in women compared to men worldwide. This difference is mainly attributable to menstruation, which likely reduces serum concentration in women by 18 % for PFOA and 29 % for PFOS [62].

While for PFOA the reduction of 18 % explained only in part the lower (−52 %) average serum concentration observed in the female subjects (Annex F), for PFOS, the smaller value observed in women (−29 %) was entirely explained by the percentage attributed to menstruation. Therefore, the predicted average PFOS concentration decreased by this percentage was taken as a reference value to derive further modifications implemented in the ML2 for women, named the “modified Loccisano 29 model” (ML29) -Annex D-. The adjustments were implemented in the code of the ML2 model for PFOS only since the predicted PFOA concentrations for women living in the B-red area were lower than the observed concentrations (section 3.8 and Annex F).

Therefore, the equation describing the plasma compartment in the ML2 code of women was adjusted to obtain a decrease of 29 % in the average predicted concentrations. The adjustment consisted of adding a term to the equation describing the mass of PFOS in time in the plasma compartment. The added term: “−N∙CA∙QCP” (μg/h), was named the “menstruation term”. N is the “menstruation coefficient” (dimensionless), CA is the PFOS concentration in plasma (μg/L) and QCP is the plasma flow in humans (L/h).

The value of the N coefficient was predicted through an iterative process based on running the simulations to get 29 % less of the original predicted value obtained with the ML2. The added term represents the PFOS loss due to menstruation in fertile women. The term: “−N∙QCP” (L/h) represents the average plasma flux lost every hour due to the menstrual cycle by a woman during the exposure period.

Therefore, the equation for the plasma compartment in the model was rewritten as follows (equation 1).

$$APlas^{\prime }=QF∙CF∙FreeF+\left(QL+QG\right)∙CL∙FreeL+QR∙CR∙FreeR+QSk∙CSk∙FreeSk+QK∙CK∙FreeK-QCP∙CA∙Free-N∙CA∙QCP$$

Equation 1 APlas’ = mass of PFOS in time in the plasma compartment (μg/h); QF/QL/QG/QR/QSk/QK = plasma flow to fat/liver/gut/rest of tissues/skin/kidney (L/h); CF/CL/CR/CSk/CK/CA = concentration of PFOS in fat/liver/rest of tissues/skin/kidney/plasma (μg/L); FreeF/FreeL/FreeR/FreeSk/FreeK/Free = free fraction of PFOS in fat/liver/rest of tissues/skin/kidney/plasma (dimensionless); QCP = plasma flow (L/h); N = menstruation coefficient (dimensionless).

The iteration process used to determine the values of parameter N (Annex C) for every municipality was implemented with B-M software, and the uncertainty associated with the prediction was derived from the precision of the PFOS serum concentration values (=0.1 ng/mL).

Starting from the value of N, the average blood flux and the average plasma flux lost during each menstruation cycle were predicted for the women of each municipality (Annex E).

#### 3.3.2 The “validity” of the menstruation term and the modified Loccisano-Verner model

The results (Annex E) obtained using this method demonstrated that the average plasma volume lost with menstruation during one cycle (corrected for fertile age) was similar (+51 %) to the value proposed by Verner et al. [47]. This discrepancy was very small, considering the large variability range of this parameter [47,63,64] and all the assumptions and uncertainties in the system of equations.

Values of N with greater physiological validity were found using first the ML2 for the total population (i.e. without the correction in the plasma compartment for women), to predict PFOS from the beginning of the exposure until 12 years of age (= beginning of the fertile age), and then the ML29, from that moment to the actual age of the female subjects, to obtain the correct value of N (predicted considering the fertile period in women). Therefore, at first the ML2 was run over the average time of exposure for women living in the municipality during the not-fertile age only and the predicted PFOS concentrations until 12 years of age were obtained (Annex E). Subsequently, those values were assumed to be the initial concentrations in the ML29 model, that was used from 12 years of age until the age at the blood sampling. In this way the correction for menstruation losses was applied only when women were in fertile age. Starting from the new menstruation coefficients for the fertile age (N_fert_) found following this method (Tab. E.5, Annex E), new corrected fluxes of plasma and blood lost with the menstrual cycle were calculated (Table 2).

In the new code, the predicted PFOS concentration in each compartment for the female subjects until 12 years of age was multiplied by the volume of the corresponding compartment to determine the initial value of the PFOS mass in the compartment. This value was used as input in the new model (Annex E).

Using the reverse process, the value of the menstruation coefficient (N_fert,V_) for the population of women in each municipality was calculated starting from the value of the average plasma volume lost per cycle proposed by Verner et al. [47] (Q_p,mc,fert,V_) using equation 2.

$$N_{fert,V}=\frac{Q_{p,mc,fert,V}}{QCC∙\left(1-Htc\right)∙{BW}^{0.75}∙24∙29.2∙1000∙\frac{\exp_{t}}{{age}_{fert}}}$$

Equation 2 N_fert,V_ = menstruation coefficient calculated starting from the average plasma concentration proposed by Verner et al. [47]; Q_p,mc,fert,V_ = average plasma volume lost per cycle proposed by Verner et al. [47]; QCC = cardiac blood output = 12.5 [L/(h*kg^0.75)], according to the original Loccisano model; Htc = hematocrit parameter (dimensionless, the value used here was the one proposed in the original Loccisano model: 0.44 (also used in the equations in Annex E), not the one proposed by Verner et al. [47]: 40; BW = body weight; exp_t_ = time of exposure for women living in that municipality; age_fert_ = fertile age in women until blood sampling (years).

Using the set of values for N_fert,V_ (Tab. E.5, Annex E), simulations with the ML29 were run to predict PFOS concentrations using Q_p,mc,fert,V_. The model implemented with these adjustments was named the “modified Loccisano-Verner model” (MLV).

### 3.4 Parameter optimization

For PFOA, optimization of K_t_ and T_mc_ was conducted exclusively for women, as simulated values deviated significantly from observed ones in men, rendering parameter values meaningless. Moreover, optimization was focused on the B-Red Area due to higher result accuracy.

Initially, using B-M software, the K_t_ parameter was adjusted through an iterative process while keeping T_mc_ constant.

Subsequently, the reverse was performed, and finally the two parameters were adjusted together. This process yielded not only adjusted values but also a parameter value with one parameter considered correct, providing insight into the range of variation.

For PFOS, parameter adjustment in the MLV rather than in the ML29 was chosen based on the greater accuracy of the N_fert,V_ parameter, derived from more precise estimates of blood volume lost per cycle. Additionally, first-order elimination rate parameter values in the Thompson model were calculated for different municipalities. (Annex F).

The results of the optimization procedure for the ML2 and MLV models showed a slightly greater value for the T_mc_ parameter (12854 μg/h/kg^0.75^) and a slightly lower value for the K_t_ parameter (48.0 μg/L) for PFOA with respect to their initial values (10000 μg/h/kg^0.75^ and 55.0 μg/L, respectively). In contrast, for PFOS, the new value of the T_mc_ parameter was lower than the initial value (3270 μg/h/kg^0.7^) for both men (3095 μg/h/kg^0.7^) and women (2577 μg/h/kg^0.7^), while the value of K_t_ was greater (25.0 μg/L for men and 33.0 μg/L for women, with respect to 23.0 μg/L). To understand the influence of K_t_ and T_mc_ on the final results, the variation in the concentration estimated by the ML2 model for PFOA in women and PFOS in men, and by the MLV model for PFOS in women, was calculated with a 1 % increase in the chosen parameter. A significant influence of both parameters on the concentration estimates of both PFAS was observed, particularly for the MLV model's estimate of PFOS in women (−1.92 % for K_t_ and +2.19 % for T_mc_) (Annex F). The average of the elimination rate parameters of the Thompson model that best fit the observed data was 3.3E-04 [day^−1^] for PFOA and 7.0E-04 [day^−1^] for PFOS and showed a wide range of values from one municipality to another. (Annex F).

### 3.5 Individual-level analysis

The individual-level analysis involved comparing predicted and observed PFOA serum concentrations through statistical analysis using the Adapted Loccisano model to test its predictive ability as the subjects’ physical characteristics, habits and exposure parameters changed.

The analysis was conducted for the total population, as well as separately for the following groups: men, women, individuals from the A-red area, and those from the B-red area.

For PFOS, an individual-level analysis was unnecessary since the standard deviation of the PFOS concentrations observed in the environmental matrices was comparable to the value of the uncertainty associated with the predicted PFOS serum concentrations.

#### 3.5.1 Predicted vs observed PFOA serum concentrations in the most likely scenario

**Fig. 2:**
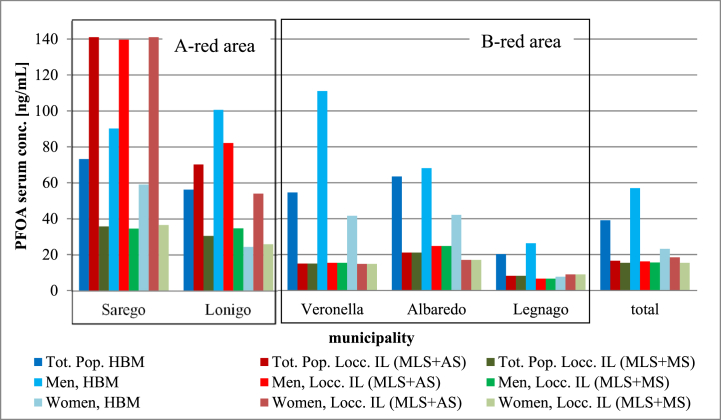
PFOA serum concentrations observed (HBM) vs predicted with the Adapted Loccisano model (Locc. IL) for each municipality, for total population (Tot.Pop), for men and for women at the individual level: median. Combined scenarios: most likely scenario + average scenario (MLS + AS) and most likely scenario + median scenario (MLS + MS).

The predicted median and average PFOA serum concentrations were lower than those observed in every municipality using the MLS + MS scenario but higher in the A-red area when adopting the MLS + AS scenario. The median was underestimated for the total population (−58/-61 %), for men (−72/-73 %) and for women (−20/-34 %). (Fig. 2 and Annex F).

In every scenario, the standard deviations were lower for the predicted values than for the observed ones.

The observed and predicted PFOA serum concentrations were not normally distributed according to the outputs of the Shapiro-Wilk test. Therefore, the Wilcoxon rank-sum WRS test was applied to compare the distributions of the data.

The null hypothesis of the WRS test with continuity correction for paired samples (i.e. predicted and observed distributions from the same population) was rejected (significance level = 0.05) for the total population (p-value = 1.5E-03), for the A-red area (p-value = 4.7E-02), for the B-red area (p-value = 1.9E-12) and for men (p-value = 3.5E-07), but not for women (p-value = 0.30).

For the MLS + MS the correlation (Pearson) was medium/low for the total population (0.39) for men (0.45) and for women (0.37) but very low (0.18) for the B-red area, where the exposure assessment was very accurate. (Annex F).

For the A-red area, the results obtained using the MLS + MS scenario were quite different from those obtained with the MLS + AS scenario, since in Sarego and Lonigo the average of the distribution of PFOA concentrations in groundwater over time was much greater than the median.

#### 3.5.2 Comparison between the different scenarios

The results were extremely different between the scenarios created for the subjects in the A-red area due to the large difference in PFOA intake. The smallest deviation from the observed average PFOA serum concentration for the A-red area was found when using the MLS + AS scenario (+44 % for Sarego and +2 % for Lonigo) and the largest difference when adopting the WCS + AS (+227 % for Sarego and +118 % for Lonigo). In contrast, for Legnago, the average concentrations predicted for the combined scenarios were similar (between 9.6 and 8.0 ng/mL) and they all underestimated the observed average values (between −70 % and −75 %).

In some cases, the observed data were higher than the values predicted for the WCS + AS scenario or lower than those predicted for the BCS + AS (Fig. F.3, Annex F), confirming the large variability in the observed concentrations when using the individual data, even wider than the range of uncertainty of the predicted values created using the different scenarios. Moreover, the observed average PFOA serum concentration ranged between the values predicted for the BCS + AS and WCS + AS scenarios, for every municipality, for the total population, for men and for women. (Annex F).

#### 3.5.3 Analysis of the population subsample

The influence of age and duration of exposure was assessed through individual-level analysis using a subset of the studied population. PFOA serum concentrations in individuals older than 20 years who had resided in the contaminated area for more than 10 years were compared with those of the total population. The selected subset (105 subjects) included a similar number of men and women and of people living in the two red areas. No substantial differences (<10 %) were identified in both the observed and predicted PFOA serum concentrations between adults (>20 years) and adolescents (14–20 years) or between subjects exposed for a long (>10 years) or short (<10 years) time. This was true for both sexes and both areas. The errors ranged from 1.8 % to 9.6 % for the observed data and from 5.6 % to 7.8 % for the predicted values. (Annex F).

### 3.6 Aggregate-level analysis

The aggregate-level analysis aimed to compare the observed average and median concentrations of PFAS with those predicted by the TK models. This analysis sought to determine the accuracy of the models by establishing a ranking.

In all aggregate-level analyses, the values related to the total population, males, females, A-red area, and B-red area were obtained by calculating the weighted average based on the number of residents in each municipality.

#### 3.6.1 PFOA

All models underestimated the average of the observed PFOA serum concentrations for all municipalities (from −31 % of the ML2 to −62 % of the Bartell model, for the total population) when using the MLS + MS. Additionally, the observed median was underestimated by all models with the exception of a slight overestimation by the ML2 for the A-red area (+11 %) and the total population (+2 %). The ML2 and Bartell models provided the most and least accurate result, respectively. The error over the total population was similar for all models (ranging from −40 % to −44 %, when compared to the observed median), except for the ML2, and was greater for the B-red area (−24 %, for the ML2).

For the A-red area, the results obtained from the one-compartment models were slightly more accurate than that from the Adapted Loccisano model, while the opposite was observed for the B-red area.

**Fig. 3:**
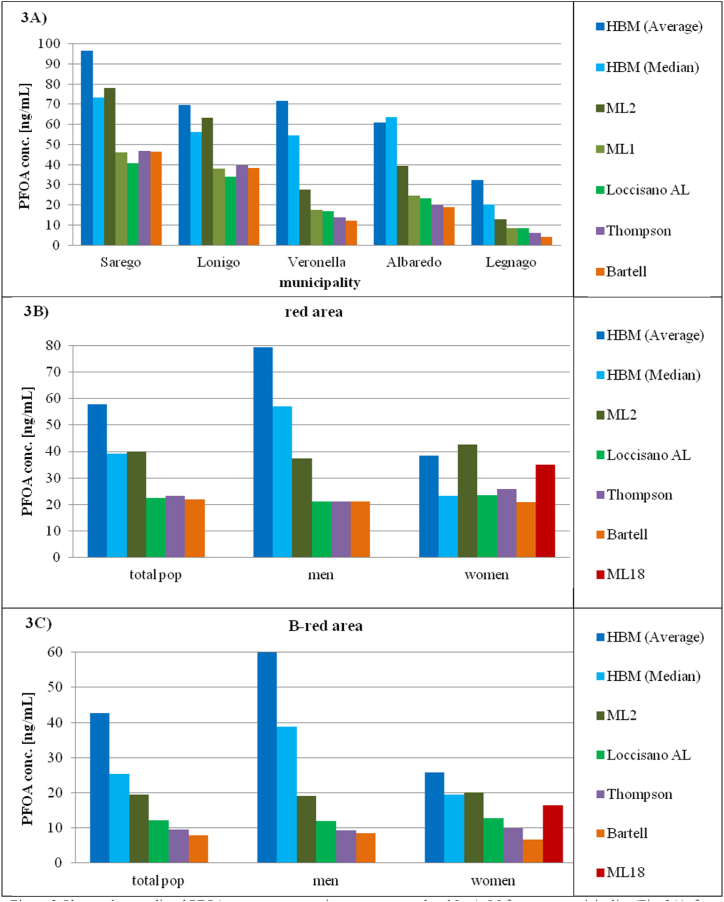
Observed vs predicted PFOA serum concentrations at aggregate level [ng/mL] for every municipality (Fig. 3A), for total population, men and women of the red area (Fig. 3B) and for the total population, men and women of the B-red area (Fig. 3C). HBM = Human Bio-monitoring study, ML2 = Modified Loccisano Version 2, ML1 = Modified Loccisano Version 1, ML18 = ML2 minus 18 %, Loccisano AL = adapted Loccisano model used at the aggregate level, Thompson = Thompson model, Bartell = Bartell model. Using the ML2 model, a significant decrease in error relative to the observed values was noted, while the ML1 model produced results very similar to those of the Loccisano, Thompson, and Bartell models across all studied municipalities when sex differences were not considered (Fig. 3A). PFAS serum concentrations were estimated more accurately in women than in men in both the red areas (Fig. 3B and C), and the ML2 and ML18 models were closer to the observed median in the B-red area (Fig. 3C).

The deviation from the observed median was greater for the B-red area (max: −69 % with Bartell, min: −24 % with the ML2). (Fig. 3 and Annex F).

The error was greater for men in every municipality but the results were similar to those for the total population. The ML2 was the model that showed the most accurate predicted values (−53 % with respect to the observed average), with the best results for the A-red area (−40 %) and for the B-red area (−68 %), while all the other models provided less accurate results (average error: −73 %). In the B-red area, the error was greater when using Bartell (−86 %) and slightly lower using the Adapted Loccisano model (−80 %). In contrast, the one-compartment models predicted the male subjects of the A-red area slightly better than the Adapted Loccisano model. The same results were obtained when comparing the predicted values to the observed median, but the errors were smaller. (Annex F).

For women, all the models, except the ML2, predicted an average PFOA concentration lower than the observed value but the extent of the underestimation was smaller than that found for the total population. The ML2 slightly overestimated the measured values (+11 %), but this result was derived from a greater overestimation for the female subjects in the A-red area (+30 %) and an underestimation for those living in the B-red area (−22 %). When menstruation losses were considered (−18 %), the deviation from the observed value decreased (−9%), but this value was derived from an overestimation for the A-red area (+7 %) and an underestimation for the B-red area (−36 %).

The Bartell model produced the least accurate output (−46 %), especially for the B-red area (−75 %), while the Adapted Loccisano and Thompson models provided very similar results (−39 % and −33 %, respectively) and showed a lower accuracy for the B-red area (−51 % and −61 %, respectively).

The observed median was overestimated by all models (max: +84 %, ML2; min: +1 %, Adapted Loccisano) except for the Bartell model (−10 %), while when considering the B-red area it was underestimated by all models (max: −66 %, Bartell), except for the ML2 model (+3 %).

#### 3.6.2 PFOS

**Fig. 4:**
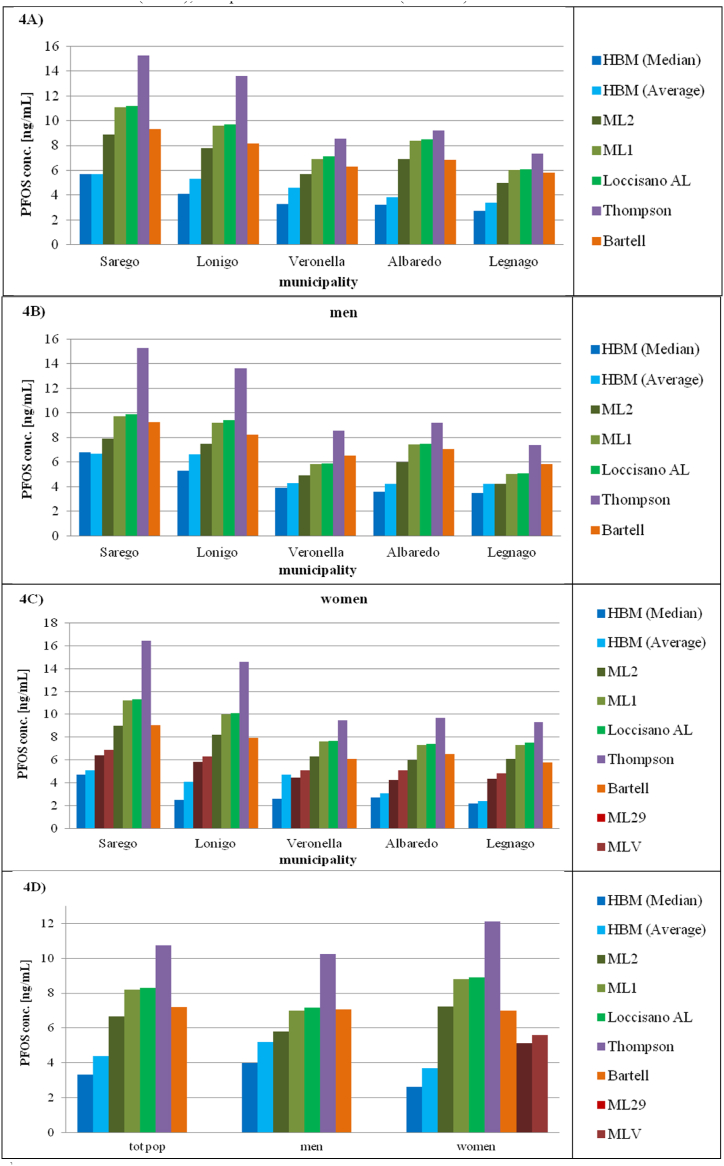
Observed vs predicted PFOS serum concentration at the aggregate level in the studied municipalities for total population (Fig. 4A), men (Fig. 4B) and women (Fig. 4C) [ng/mL]. HBM = Human Bio-monitoring study, ML29 = Modified Loccisano 29 %, MLV = Modified Loccisano-Verner, ML2 = Modified Loccisano Version 2, ML1 = Modified Loccisano Version 1, Loccisano AL = Adapted Loccisano model used at the aggregate level, Thompson = Thompson model, Bartell = Bartell model. The ML2 model yielded the most accurate predicted values across all studied municipalities, while the Bartell model produced more accurate results than the Thompson model when sex differences were not accounted for (Fig. 4A). PFOS serum concentrations were slightly better predicted for men by all models (Fig. 4B, C, and 4D). The MLV model provided the best results for women, significantly improving prediction accuracy compared to the ML2 model (Fig. 4C and D).

All models overestimated the average serum concentrations of PFOS observed in the HBM study (Fig. 4).

The ML2 provided the most accurate predicted values (+50 %, with respect to the observed average) while the Thompson model provided the least accurate result (+134 %). The error was between +82 % and +85 % when using the Loccisano-based models, while the Bartell model predicted the observed concentrations more accurately (+63 %). The error was slightly greater for the A-red area (+88 %) than for the B-red area (+79 %), except for the Bartell model. (Annex F).

The overestimation for men was lower than that for the total population but similar considerations can be made. The ML2 provided the most accurate predicted values (+12 % with respect to the observed average), for both the A-red area (+15 %) and the B-red area (+10 %). All models provided better results for the B-red area, except for Bartell. The Loccisano-based models and Bartell model provided similar results (+35 %, +38 %, +39 %) while the Thompson model predicted higher concentrations (+96 %), especially in the A-red area.

The overestimation for women was greater than that for the total population for all the models. The ML29 provided the best results (+47 % with respect to the observed average). The MLV was slightly less precise than the ML29 (+63 %). The only underestimation of the observed average (but not of the median) was registered for Veronella, using the ML29 (−4%). The largest error (+242 %) was observed using the Thompson model, particularly for Legnago (+288 %).

In conclusion, the most accurate models for predicting PFOS serum concentration were the ML2 for men and ML29 for women, while the greatest errors occurred using the Thompson model, for both sexes. All the models predicted better PFOS concentrations in men.

#### 3.6.3 Analysis of the PFAS serum concentration trend over time and the contribution of exposure routes to the total uptake

The predicted PFAS serum concentration exhibited distinct temporal trends across municipalities and scenarios. On average, its value at steady state significantly differed from the predicted peak, with a clear difference between PFOA and PFOS (49 % and 84 % respectively). However, the disparity between the serum PFAS concentrations at the peak and at the time of blood sampling was minimal (13 % for PFOA and 5 % for PFOS).

In addition, the predicted contributions of PFAS serum concentrations from groundwater, tap water and food to the total uptake were investigated by modifying the code of the ML2 model to consider only the specific contribution attributable to each route of exposure.

The estimated contribution of each exposure route differed substantially between PFOA and PFOS, as well as between the two areas, while remaining consistent between genders.

Considering the B-red area only, food emerged as the primary route of exposure for both PFAS.

Conversely, in the A-red area, for PFOA, the principal contribution was attributed to groundwater. (Annex F).

#### 3.6.4 Individual vs aggregate-level analysis

The Adapted Loccisano model yielded nearly identical average serum concentrations of PFOA when utilizing individual or aggregate data (52.4 vs 46.5 ng/mL, respectively). Interestingly, the deviation from the observed value when employing the Loccisano model with aggregate data from the total population (−9.6 %) was even lower than that obtained using the model at the individual level (−19.8 %). (Annex F).

### 3.7 Limitations and solved issues related to water exposure assessment

In conducting the exposure assessment, three significant challenges were encountered and partially resolved through the formulation of exposure scenarios (section 2.2.1.1).

First, inaccuracies were identified in the questionnaire administered in the context of the HBM study, with certain pertinent inquiries -e.g. tea consumption prepared using tap water-being omitted.

Second, the initial questionnaire, which was conducted before August 2018, lacked specificity in its inquiries regarding water usage timeframes. Conversely, the subsequent questionnaire, administered post-August 2018, referred to periods preceding the installation of GAC filters. This resulted in an information gap regarding water usage post-GAC filter installation.

Finally, the reliability of the responses regarding private well ownership led to concerns. Given that most private wells were not reported to relevant authorities, many subjects may have denied ownership of them.

Additionally, concerns arose regarding the highest PFAS concentrations detected in both tap water and groundwater (Annex A). These data were not treated as outliers, as their elevated values likely stemmed from samples collected at different locations and/or time periods. Therefore, the primary factors considered were the spatial and temporal variability of PFAS concentrations in groundwater, along with temporal variability in tap water. These assumptions were corroborated by studies on the temporal evolution of contaminated plumes [13,14,36] and the trend of PFAS concentrations in raw water (Annex A).

The concentration trends in groundwater and raw water justified the end date of the first exposure period related to tap water (see Annex A), while the concentration trends in treated water justified the start and end dates of subsequent exposure periods (see section 2.2.1 and Annex A). Notably, for PFOS, the accuracy of the exposure assessment was fairly high, as for PFOA in the B-red area, while it was lower for PFOA in the A-red area. Indeed, all subjects within the same municipality were exposed to identical PFAS concentrations in tap water. Conversely, in the A-red area, significant disparities in PFOA concentrations were observed in groundwater across different locations within the same municipality. This large spatial variability of PFOA translated into substantial differences in exposure among subjects drawing from different private wells, even within the same municipality. As a result, for PFOA, a moderate to high level of uncertainty characterized exposure scenarios for the A-red area, while a low level of uncertainty was associated with exposure assessments for subjects in the B-red area (Tab. A.14, Annex A). Nonetheless, while spatial variability in PFOA concentrations in groundwater posed challenges in individual-level analyses, it was much less impactful in analyses utilizing aggregate data, where municipality-wide averages or medians were employed to represent subjects across various locations within the municipality. The impact of the assumptions in section 2.2.1.1 on the result is reflected in the differences observed in the predicted serum concentrations across the combined scenarios (Fig. F.3, Annex F).

Another limitation of the study concerns the comparison between municipalities. Although the selected subjects are fairly representative of the population of the entire municipality, the median PFAS serum concentration of a city's population should be adjusted for potential predictors such as age, sex, and blood sampling date. However, these variables were taken into account for the subjects involved in this study (section 2.1) to reduce the error.

It is important to note that, prior to 2013, PFASs were not measured in Italian waters. Following the detection of these contaminants and the increasing scientific evidence of their potential health effects, progressively stricter concentration limits for PFASs in drinking water have been implemented.

In 2013, despite the drinking water and surface water in the study area being considered of good quality, PFAS concentrations were extremely high. This is because generally studies assessing the quality of surface waters [[65], [66], [67]] using the Water Quality Index or similar take into account numerous parameters, but not the concentration of PFASs, as these do not affect the monitored physico-chemical parameters. Therefore, it would be beneficial to understand how to incorporate PFASs concentrations into water quality indices.

### 3.8 Considerations about TK models

Before discussing the results, it seems appropriate to highlight a few considerations on the rationale behind selecting the specific set of parameters modified in the PBPK model. This was primarily driven by the selected parameters significant variability observed in humans, which is difficult to explain, resulting in numerous divergent values (still under discussion) reported in the literature (Annex C). For example, the value of T_mc_ for PFOA in the original Loccisano model corresponds to a 2.3 year half-life, while its modified value in the ML2 model corresponds to a 3.8 year half-life. Both values appear plausible, but 2.3 years is the smallest value found in the literature for PFOA half-life [57].

Other parameters in the model present less uncertainty (e.g., parameters whose values were collected in the questionnaire administered as part of the HBM study, such as body weight or food intake) and/or have fewer alternative values available in the literature (e.g., physical parameters).

Furthermore, we focused on these parameters because they model specific characteristics of PFAS. Specifically, they describe the renal resorption process, the free concentration of PFAS in the blood, and their distribution across various tissues. Additionally, these parameters are known to have high sensitivity [51,57], and their impact on the results was also examined in this study.

Indeed, an indication of the impact of this set of parameters on the results is provided by the difference between the outcomes when using the modified model compared to the previous version of the model (Results). A minimal influence of the partition coefficients proposed by Fabrega et al. [50] was observed in the ML1 model, while a significant influence of the parameters modified in the ML2 model was expected from previous sensitivity analyses [51].

Regarding the individual parameters, the impact of the K_t_ and T_mc_ coefficients was analyzed during the optimization phase (Section 3.4).

Moreover, the significant impact of the N coefficient on the results is evident, as even a minimal variation in this parameter leads to a substantial change in the final outcomes of the MLV and ML29 models (Results; Tab. E.4 and E.5, Annex E). The Loccisano model was chosen among the various multi-compartment PBTK models he because it predicts PFAS uptake in many human tissues through a system of differential equations, that account for the renal resorption process, which plays an important role in accurately predicting the PFAS pathway in the human body [25]. Moreover, it has been shown to provide reliable predictions of PFOA plasma concentrations and has been evaluated using extensive data under different exposure conditions; making it the most appropriate model to use [57].

Analyzing the model comparison, the obtained results hold greater validity within the study's conditions (i.e. population exposed to high PFOA and medium-low PFOS serum concentrations, diet as the primary route of exposure and a post-ban context). Nevertheless, the broader applicability of these findings to different scenarios is assured by the type of analyses performed, the values for the model parameters sourced from the literature, and the methodological consistency with other studies (section 1).

Additionally, utilizing the Loccisano-based models offers a significant advantage over the Thompson model, as the former provides extensive information in the model output, including the trend of PFAS serum concentration over time. While the Bartell model also offers insight into this information, it necessitates constant exposure. Therefore, when the analysis aims to predict PFAS concentrations over time, especially in scenarios with non-constant exposure, Loccisano-based models are highly recommended.

Furthermore, it is crucial to emphasize that only Loccisano-based models enable the prediction of PFAS concentrations in various tissues. However, they are also obtainable using one-compartment models to predict serum concentration and subsequently multiplying by partition coefficients available in the literature.

In conclusion, the values of the predicted PFAS concentrations were strongly influenced by the PFAS concentrations in the water, water intake and body weight of the subjects. This underscores the importance of analyzing population characteristics and ensuring the precision of the exposure assessment.

For PFOA, the ML2 model exhibited a slight overestimation of the average observed concentration for all women, yet a slight underestimation was observed for those in the B-red area, where the data accuracy is higher. Therefore, for PFOA, the implementation of a model specific for women with menstruation correction was deemed pointless since such modification would have increased the error of the model output for women in the B-red area.

The slightly larger deviation between predicted and observed values in Veronella and Albaredo compared to the other municipalities may not be attributable to the model code, but rather to the collection of water samples (i.e. it could be linked to spatial variability in PFOA concentration). In fact, the observed PFOA concentrations in contaminated water, used as model input, mirrored the trend predicted by the outputs generated, while the observed PFOA serum concentrations did not. Additionally, the smaller sample size and the significantly different number of men and women likely amplified the importance of the error.

However, this discrepancy did not significantly impact the final results, despite resulting in a slightly reduced (for PFOA) and slightly increased (for PFOS) deviation of the output values from the observed data.

It is noteworthy that the Loccisano-based models were modified to account for the decline in PFAS concentration in tap water in the years leading up to blood sampling, whereas the Thompson and Bartell models required a constant exposure over time. This fact led to less accurate and detailed exposure data being input into the two one-compartment models, resulting in a slight overestimation of PFAS concentration intake. However, this approximation did not significantly affect the final results, despite causing slightly reduced (for PFOA) and slightly increased (for PFOS) deviation of the output values from the observed data.

The errors in the results predicted by the Adapted Loccisano and Bartell models were comparable, yet the Bartell model necessitated less time, effort, and expertise to configure the simulations. Moreover, the Bartell model requires minimal expertise to run simulations, owing to the implementation of the online serum calculator for PFASs [46]. Therefore, the slightly superior performance of the Adapted Loccisano model observed only for PFOA does not justify the increased time spent learning to set up simulations and the efforts to improve input data accuracy.

#### 3.8.1 Individual-vs aggregate-analysis

The decision to conduct individual-level analysis hinges on the availability of high-quality input data derived from accurate exposure assessments for each subject. In the current case study, this criterion was met for the residents of the B-red area. However, the individual-level approach proved to be unnecessarily laborious and time-consuming. In fact, the aggregate-level analysis demonstrated comparable reliability and accuracy, particularly when examining aggregate indicators, which were well predicted by both approaches. Furthermore, the limited correlation observed through statistical analysis and the discrepancies between the HBM data and those predicted by the Adapted Loccisano model in the BCS and WCS underscored the challenges in accurately predicting the high inter-individual variability in PFOA serum concentrations using Loccisano-based models.

#### 3.8.2 Considerations on sex differences

In alignment with the literature [[68], [69], [70], [71], [72], [73]], this study revealed higher PFAS serum concentrations in men.

Interestingly, while a higher intake rate of contaminated water would likely result in increased PFAS uptake, a greater body weight would lead to a lower amount of PFAS per kilogram of body weight. Consequently, the higher PFAS serum concentrations observed in men cannot be solely attributed to a higher water intake rate, as this factor may be compensated by their greater body weight.

The sex difference in concentration could not be entirely explained by menstruation [62,63]. Although the specific causes were not investigated in our study, if we assume that the reduction reported by Gomis et al. [62] is applicable to women in the Veneto region, the observed sex difference in average PFOA serum concentration (−52 %) cannot be solely attributed to menstruation. Conversely, the 29 % lower observed average PFOS concentration for women aligns with the percentage associated with menstruation in the Gomis et al. [62] study. This finding implies that the actual sex difference attributed to menstruation may be less than suggested. Nonetheless, the identified difference in PFAS serum concentrations between men and women of fertile age is noteworthy for various populations, emphasizing the importance for modellers to consider sex differences in PFAS-specific models.

## 4 Conclusions

The primary objective of this study was to assess the exposure of individuals exposed to PFAS contamination and to evaluate the accuracy and cost-effectiveness of various PFAS-specific TK models in predicting observed data.

Additionally, specific parameters and a differential equation of a complex multi-compartment model were adjusted to obtain more accurate predictions of the observed data.

The current study demonstrated promising results achieved through the application of both empirical TK and PBTK models in predicting PFAS serum concentrations in subjects with long-term exposure to contaminated drinking water and food.

For PFOA, all models predicted slightly better results for women and residents of the A-red area, while for PFOS, better results were obtained for men. Considering the entire population, the observed concentrations were underestimated by all tested models for PFOA, whereas for PFOS, they were overestimated.

Notably, the use of modified models led to highly accurate predictions for both PFAS. Specifically, for both PFAS, the ML2 model emerged as the most accurate, except for PFOS in women, that was predicted better by the ML29 and MLV models. However, taking into account the potential underestimation of actual values, particularly for men, the Bartell model proved to be a practical solution for predicting PFAS serum concentrations. In fact, this online serum calculator demonstrated user-friendliness, time efficiency, and reasonable accuracy. Nevertheless, when exposure varies significantly over time and its end is distant in time (>2 years), the Bartell model's reliability diminishes, warranting the use of the PBTK models that incorporate renal resorption processes. Finally, only marginal improvements in the accuracy of the results were observed when using the ML1 model instead of the Adapted Loccisano model.

In light of these findings, it becomes evident that environmental protection agencies and health authorities may find it advantageous to employ the tested models at the aggregate level rather than conducting more invasive and expensive screening campaigns as an initial step in HBM studies. This holds particularly true for PFOA in women and PFOS in both sexes when using the modified models. However, for PFOA in men, the errors produced by all the tested models were deemed too substantial to consider them sufficiently reliable.

In the near future, the availability of more precise data on subject locations and PFAS concentrations in groundwater is anticipated. Consequently, the accuracy of exposure assessments could improve, enabling a more comprehensive analysis.

## Ethical statement

Our study does not directly involve human subjects, and the Veneto Region and University of Padua provided us with the human data used in this study in an anonymous format. In addition, these data were collected as part of a human bio-monitoring study accurately described by Pitter et al. [35]. As such, no ethical approval was required for the use of these data.

## Data availability statement

Environmental data are available here: https://www.arpa.veneto.it/dati-ambientali/open-data/idrosfera/concentrazione-di-sostanze-perfluoroalchiliche-pfas-nelle-acque-prelevate-da-arpav while the ownership of human data belongs to the Veneto Region.

Unfortunately, due to Italian regulations regarding personal data protection, it is not possible to share individual health data outside of the project, nor to make such data publicly available. Aggregate health data are included in the article and supp. material or referenced in article.

## Funding

This work was supported by the Consortium for Healthcare Research (10.13039/100018331CORIS).

## CRediT authorship contribution statement

**L. Vaccari:** Writing – original draft, Visualization, Validation, Software, Methodology, Formal analysis, Data curation, Conceptualization. **A. Ranzi:** Writing – review & editing, Supervision, Resources, Methodology, Investigation, Conceptualization. **C. Canova:** Investigation, Data curation. **G. Ghermandi:** Supervision, Resources, Investigation. **S. Giannini:** Formal analysis, Data curation. **G. Pitter:** Investigation, Data curation. **F. Russo:** Supervision, Resources. **J. Stefanelli:** Investigation, Data curation. **S. Teggi:** Supervision, Resources, Conceptualization. **A. Vantini:** Investigation, Data curation. **M.Z. Jeddi:** Writing – review & editing, Visualization, Methodology, Conceptualization. **T. Fletcher:** Writing – review & editing, Supervision, Methodology, Conceptualization. **A. Colacci:** Writing – review & editing, Supervision, Resources, Project administration, Methodology, Funding acquisition.

## Declaration of generative AI and AI-assisted technologies in the writing process

During the preparation of this work the authors used ChatGPT (https://chat.openai.com/), developed by Open AI, in order to improve language. After using this tool, the authors reviewed and edited the content as needed and take full responsibility for the content of the publication.

## Declaration of competing interest

The authors declare that they have no known competing financial interests or personal relationships that could have appeared to influence the work reported in this paper.
