# Supplementary material for: Reliability of toxicokinetic modelling for PFAS exposure assessment in contaminated water in northern Italy
Source: Heliyon. 2024 Jul 31;10(15):e35288. doi: 10.1016/j.heliyon.2024.e35288 (PMC11334853; doi:10.1016/j.heliyon.2024.e35288)
Supplement: Multimedia component 1 [file mmc1.docx]

## **POPULATION ANALYSIS**

**Population characteristics and statistic tests:**

| **summary of population characteristics (average value ± standard deviation)** | | | | | | | |
| --- | --- | --- | --- | --- | --- | --- | --- |
|  | **C-PFOA (ng/mL)** | **C-PFOS (ng/mL)** | **Exp_t_ (years)** | **Age (years)** | **A-WIR (L/day)** | **A-BW (kg)** | **N** |
| **M** | 78.0 ± 71.4 (m: 57.0) | 5.2 ± 3.3 (m: 4.0) | 18.7 ± 7.7 | 26 ± 8 | 1.9 ± 0.7 | 77 ± 16 | 88 |
| **W** | 38.5 ± 44.4 (m: 23.2) | 3.7 ± 3.4 (m: 2.6) | 17.7 ± 8.9 | 27 ± 7 | 1.5 ± 0.7 | 60 ± 11 | 91 |
| **Tot pop** | 57.9 ± 62.3 (m: 39.1) | 4.4 ± 3.4 (m: 3.3) | 18.2 ± 8.3 | 26 ± 8 | 1.7 ± 0.8 | 69 ± 16 | 179 |
| **A-red area** | 78.6 ± 67.0 (m: 61.9) | 5.4± 4.1 (m: 4.6) | 17.7 ± 8.7 | 26 ± 8 | 1.6 ± 0.8 | 66 ± 16 | 76 |
| **B-red area** | 42.6 ± 47.6 (m: 32.3) | 3.6± 2.5 (m: 2.9) | 18.6 ± 8.1 | 27 ± 7 | 1.7 ± 0.7 | 70 ± 16 | 103 |

Table 1A Summary of population characteristics (average value ± standard deviation) for men (M), women (W) and total population (tot pop). C-PFOA = average observed PFOA serum concentration, C-PFOS = average observed PFOS serum concentration, Exp_t_ = exposure time, A-WI = average water intake, A-BW = average body weight, N = number of subjects. For PFAS serum concentrations also the median is reported (m).

| **Municipality** | **C-PFOA (ng/mL)** | | **C-PFOS (ng/mL)** | | **N** |
| --- | --- | --- | --- | --- | --- |
|  | **A.V.** | **median** | **A.V.** | **median** |  |
| **Sarego** | 96.7 | 73.3 | 5.7 | 5.7 | 25 |
| **Lonigo** | 69.7 | 56.3 | 5.3 | 4.1 | 51 |
| **Legnago** | 32.2 | 20.3 | 3.4 | 2.7 | 72 |
| **Veronella** | 71.6 | 54.6 | 4.6 | 3.3 | 15 |
| **Albaredo d’Adige** | 60.8 | 63.5 | 3.8 | 3.2 | 17 |

Table 2A PFAS serum concentrations (average value and median) for every municipality. C-PFOA = average observed PFOA serum concentration, C-PFOS = average observed PFOS serum concentration. N = number of subjects.

|  | **sex** | | **area** | |
| --- | --- | --- | --- | --- |
|  | **W** | **p-value** | **W** | **p-value** |
| **C-PFOA** | 2269 | 5.598E-07 | 5360 | 2.46E-05 |
| **C-PFOS** | 2406 | 4.017E-06 | 5038 | 1.04E-03 |

Table 3A Outputs of the Wilcoxon rank sum test with continuity correction run to compare the group of men versus the group of women (sex) and the group of subjects living in the A-red area versus the group of subjects living in the B-red area (area) for observed PFAS serum concentrations. W = statistic, p-value.

|  | age | exp_t_ | Water intake | weight |
| --- | --- | --- | --- | --- |
| p-value | 0.29 | 0.41 | 2.85E-04 | 5.92E-15 |
| Null hypothesis | Not rejected | Not rejected | Rejected | Rejected |

Table 4A outputs of the t-tests run to compare the group of men versus the group of women for age, exposure time, (expt), water intake and weight.

|  | age | exp_t_ | Water intake | weight |
| --- | --- | --- | --- | --- |
| p-value | 0.68 | 0.47 | 0.26 | 0.10 |
| Null hypothesis | Not rejected | Not rejected | Not rejected | Not rejected |

Table 5A outputs of the t-tests run to compare the group of subjects living in the A-red area versus the group of subjects living in the B-red area for age, exposure time, (expt), water intake and weight.

## **Exposure assessment**

**Time periods chosen in tap water and groundwater:**

The chosen time periods for the different municipalities and the corresponding average PFOA concentration values are reported in the following tables:

| **Municipality** | **P1** | **P2** | **P3** | **P4** | **First sample-last sample collected > 5** |
| --- | --- | --- | --- | --- | --- |
| **Sarego** | BD-7/14 | 8/14-10/17 | 11/17-BS |  | 8/13-10/17 |
| **Lonigo** | BD-2/14 | 3/14-4/16 | 5/16-10/17 | 11/17-BS | 8/13-10/17 |
| **Legnago** | BD-11/16 | 12/16-BS |  |  | 11/16-11/16 |
| **Veronella** | BD-10/14 | 11/14-10/17 | 11/17-BS |  | 7/13-8/17 |
| **Albaredo d’Adige** | BD-2/15 | 3/15-10/17 | 11/17-BS |  | 7/13-8/17 |

Table 6A Selected time periods (Pi) for PFOA average concentration values in tap water. BD= birth date of the subject. BS=date of the blood sampling.

| **Municipality** | **A-PFOA, P1 [ng/L]** | **A-PFOA, P2 [ng/L]** | **A-PFOA, P3 [ng/L]** | **A-PFOA, P4 [ng/L]** | **A-PFOA, (until the last value higher than 5 ng/L)** | **Number of samples collected (until the last value higher than 5 ng/L)** |
| --- | --- | --- | --- | --- | --- | --- |
| **Sarego** | 99 | 27 | 5 |  | 38 | 185 |
| **Lonigo** | 249 | 89 | 61 | 5 | 114 | 182 |
| **Legnago** | 27 | 5 |  |  | 27 | 1 |
| **Veronella** | 184 | 116 | 5 |  | 148 | 26 |
| **Albaredo d’Adige** | 255 | 124 | 5 |  | 192 | 23 |

Table 7A Average PFOA concentrations in tap water (A-PFOA) for the different time periods (Pi).

The chosen time periods for the different municipalities and the corresponding average PFOS concentration values are reported in the following table:

| **Municipality** | **P1** | **P2** | **P3** | **A-PFOS, P1 [ng/L]** | **A-PFOS, P2 [ng/L]** | **A-PFOS, P3 [ng/L]** |
| --- | --- | --- | --- | --- | --- | --- |
| **Sarego** | BD-7/14 | 8/14-BS | - | 38 | 10 | - |
| **Lonigo** | BD-2/14 | 3/14-10/17 | 11/17-BS | 37 | 25 | 18 |
| **Legnago** | BD-BS | - | - | 5 | - | - |
| **Veronella** | BD-BS | - | - | 13 | - | - |
| **Albaredo d’Adige** | BD-6/16 | 7/16-BS | - | 18 | 10 | - |

Table 8A Selected time periods (Pi) for PFOS average concentrations in tap water (A-PFOS) and average PFOS concentrations in tap water (A-PFOS) for the different time periods (Pi). BD= birth date of the subject. BS=date of the blood sampling.

**Exposure to groundwater:**

**PFOA:**

|  | **A-PFOA [ng/L]** | **M-PFOA [ng/L]** | **SD-PFOA [ng/L]** | **5-95 percentile [ng/L]** | **D_first_-D_last_** | **N** |
| --- | --- | --- | --- | --- | --- | --- |
| **Sarego** | 3754 | 680 | 5432 | 84-14886 | 7/13-10/19 | 138 |
| **Lonigo** | 1657 | 400 | 2490 | 18-6895 | 7/13-10/19 | 237 |
| **Legnago** | 19 | 10 | 20 | 10-46 | 4/16-9/19 | 118 |
| **Albaredo d’Adige** | <10 | <10 | - | - | 12/15-9/17 | 19 |

Table 9A PFOA average concentration in groundwater (A-PFOA), median (M-PFOA), standard deviation (SD-PFOA), 5-95 percentile of the distribution, date of the first-last collected sample (D_first_-D_last_) and number of samples collected (N).

**PFOS:**

|  | **A-PFOS [ng/L]** | **M-PFOS [ng/L]** | **SD-PFOS [ng/L]** | **5-95 percentile [ng/L]** | **D_first_-D_last_** | **N** |
| --- | --- | --- | --- | --- | --- | --- |
| **Sarego** | 29 | 21 | 23 | 10-83 | 7/13-10/19 | 138 |
| **Lonigo** | 37 | 37 | 23 | 10-77 | 7/13-10/19 | 237 |
| **Legnago** | <10 | <10 | - | - | 4/16-9/19 | 118 |
| **Albaredo d’Adige** | <10 | <10 | - | - | 12/15-9/17 | 19 |

Table 10A PFOS average concentration in groundwater (A-PFOS), median (M-PFOS), standard deviation (SD-PFOS), 5-95 percentile of the distribution, date of the first-last collected sample (Dfirst-Dlast) and number of samples collected (N).

**Scenarios:**

- Worst-case scenario (WCS), with the following characteristics:

water intake: 1/3 from private wells, 1/3 from tap water, 1/3 from bottled water. End of exposure to private well: at the time of the sample collection. In this scenario a change in water use after the detection of PFAS in groundwater in 2013 was not supposed to happen;

- Most likely scenario (MLS), with the following characteristics:

water intake: 1/3 from private wells, 1/3 from company water, 1/3 from bottled water. Exposure to private well was assumed to last until the end of the time period P1, when the PFAS contamination was detected and the Veneto population was informed about the risks associated with the intake of water from the private wells. So, this scenario took into account a change in water use after the detection of PFAS in groundwater in 2013. This was considered the most likely scenario;

- Best-case scenario (BCS), with the following characteristics:

No exposure to private well was considered in this scenario. Water intake was assumed to be 1/3 from tap water and 2/3 from bottled water. So, water intake (varying from subject to subject) was divided into clean (2/3 of the total) and contaminated (1/3 of the total) water intake rate.

**PFAS daily intake through drinking water:**

$${PWI}_{n}={PCTW}_{m}\cdot{TWIR}_{n}+{PCGW}_{m}\cdot{GWIR}_{n}$$

Where:

PWI_n_ = PFAS daily intake through drinking water for the n-subject [ng/day];

PCTW_m_ = PFAS concentration in tap water in the m-municipality [ng/L];

TWIR_n_ = tap water intake rate for the n-subject [L/day];

PCGW_m_ = PFAS concentration in groundwater in the m-municipality [ng/L];

GWIR_n_ = groundwater intake rate for the n-subject [L/day];

Since:

$${TWIR}_{n}={GWIR}_{n}={CWIR}_{n}=1/3\cdot{WIR}_{n}$$

Where:

CWIR_n_ = contaminated water intake rate (for each drinking water source) for the n-subject [L/day].

WIR_n_ = water daily intake rate for the n-subject [L/day]

The previous equation can also be rewritten as follow:

$${PWI}_{m}={(PCTW}_{m}+{PCGW}_{m})\cdot{CWIR}_{n}$$

$${PWI}_{m}={PCW}_{m}\cdot{CWIR}_{n}$$

$${PWI}_{m}={PCW}_{m}\cdot{1/3\cdot WIR}_{n}$$

Where:

PCW_m_ = total PFAS concentration in water (sum of PFAS concentration in tap water and PFAS concentration in groundwater) in the m-municipality [ng/L].

|  | **PFOA A-WI, tot pop [ng/day]** | **PFOA A-WI, men [ng/day]** | **PFOA A-WI, women [ng/day]** | **PFOA A-CW [ng/L]** | **A-CWIR [L/day]** | **A-WIR [L/day]** |
| --- | --- | --- | --- | --- | --- | --- |
| **Sarego** | 381.7 (MS)  1888.0 (AS) | 402.5 (MS)  1990.7 (AS) | 368.7 (MS)  1823.8 (AS) | 779 (M)  3853 (A) | 0.49 | 1.47 |
| **Lonigo** | 359.1 (MS)  1054.7 (AS) | 395.9 (MS)  1162.7 (AS) | 324.5 (MS)  953.0 (AS) | 649 (M)  1906 (A) | 0.55 | 1.66 |
| **Legnago** | 26.2 | 28.2 | 24.1 | 46 | 0.57 | 1.71 |
| **Veronella** | 98.1 | 134.9 | 79.7 | 184 | 0.53 | 1.60 |
| **Albaredo d’Adige** | 159.0 | 181.9 | 117.3 | 255 | 0.62 | 1.87 |

Table 11A PFOA average contaminated water intake rate (PFOA A-WI ) and average PFOA concentration in water (PFOA A-CW), that is the sum of PFOA concentration in tap water and PFOA concentration in groundwater, average contaminated water intake rate (A-CWIR) and average water intake rate (A-WIR). M=median, A= average value. MS = median scenario, AS = average scenario.

|  | **PFOA A-WI, tot pop [ng/day]** | **PFOA A-WI, men [ng/day]** | **PFOA A-WI, women [ng/day]** | **PFOA A-CW [ng/L]** | **A-CWIR [L/day]** | **A-WIR [L/day]** |
| --- | --- | --- | --- | --- | --- | --- |
| **Total pop. (MS)** | 188.4 | 196.3 | 182.3 | 349.9 | 0.55 | 1.67 |
| **A-red area (MS)** | 366.5 | 397.8 | 340.3 | 691.8 | 0.53 | 1.60 |
| **B-red area** | 58.3 | 69.4 | 46.9 | 100.1 | 0.57 | 1.72 |
| **Total pop. (AS)** | 594.7 | 585.9 | 608.6 | 1133.0 | 0.55 | 1.67 |
| **A-red area (AS)** | 1328.8 | 1406.2 | 1264.0 | 2546.5 | 0.53 | 1.60 |

Table 12A PFOA average contaminated water intake rate (PFOA A-WI ) and average PFOA concentration in water (PFOA A-CW), that is the sum of PFOA concentration in tap water and PFOA concentration in groundwater, average contaminated water intake rate (A-CWIR) and average water intake rate (A-WIR). MS = median scenario, AS = average scenario.

|  | **PFOS WI, tot pop [ng/day]** | **PFOS WI, men [ng/day]** | **PFOS WI, women [ng/day]** | **PFOS CW [ng/L]** | **A-CWIR** | **A-WIR** |
| --- | --- | --- | --- | --- | --- | --- |
| **Sarego** | 32.8 | 34.6 | 31.7 | 67 | 0.49 | 1.47 |
| **Lonigo** | 27.1 | 29.9 | 24.5 | 49 | 0.55 | 1.66 |
| **Legnago** | 5.7 | 6.1 | 5.2 | 10 | 0.57 | 1.71 |
| **Veronella** | 9.6 | 13.2 | 7.8 | 18 | 0.53 | 1.6 |
| **Albaredo d’Adige** | 14.3 | 16.4 | 10.6 | 23 | 0.62 | 1.87 |
| **Total pop.** | 16.7 | 17.5 | 15.9 | 30.9 | 0.55 | 1.67 |
| **A-red area** | 29.0 | 31.3 | 27.1 | 54.9 | 0.53 | 1.60 |
| **B-red area** | 7.7 | 8.9 | 6.4 | 13.3 | 0.57 | 1.72 |

Table 13A PFOS average contaminated water intake rate (PFOA A-WI ) and average PFOS concentration in water (PFOS A-CW), that is the sum of PFOS concentration in tap water and PFOS concentration in groundwater, average contaminated water intake rate (A-CWIR) and average water intake rate (A-WIR). M=median, A= average value. MS = median scenario, AS = average scenario.

**Contamination level ranking:**

|  | **Sarego** | **Lonigo** | **Legnago** | **Veronella** | **Albaredo d’Adige** |
| --- | --- | --- | --- | --- | --- |
| **Level of contamination in tap water** | 4 | 2 | 5 | 3 | 1 |
| **Level of contamination in groundwater** | 1 | 2 | 3 (low) | 4 (not detected) | 4 (not detected) |
| **Uncertainty at aggregate level** | low/medium | low/medium | low | low | low |
| **Uncertainty at individual level** | high | high | low | low | low |

Table 14A Contamination level ranking (1 is the highest and 5 is the lowest) and uncertainty levels associated with exposure to PFOA in water for the people residing in the different municipalities.

**Standard portion:**

| **Type of food** | **Standard portion** | **Unit** |
| --- | --- | --- |
| **Fruit** | 150 | g |
| **Raw vegetable** | 80 | g |
| **Cooked vegetable** | 200 | g |
| **Milk, yogurt** | 125 | mL |
| **Meat** | 100 | g |
| **Egg** | 50 | g |

Table 15A quantitative standards for portions in Italy according to the Italian society of human nutrition (SINU).

**Daily intake through food:**

The predicted PFAS intake through a food category was computed according to the following equation:

$${PIF}_{i}=\frac{DP\times PCF\times SP}{1000}$$

Where:

PIF_i_ = PFAS daily intake through food category i [ng/day];

DP = Daily portion of food category i [1/day];

PCF = PFAS concentration in food category i [ng/kg];

SP = Standard portion of food category i [g];

and 1000 is the correction factor [g/kg].

The total intake of PFAS through food was then computed for each subject as follow:

$${PTIF}_{n}=\sum_{i=1}^{5} {PIF}_{i}$$

Where:

PTIF_n_ = PFAS total daily intake through food estimated for the n-subject [ng/day].

| **Municipality** | **A-PTIF [ng/day]** | **A-PIF_fruit&veg_ [ng/day]** | **A-PIF_milk_ [ng/day]** | **A-PIF_meat_ [ng/day]** | **A-PIF_egg_ [ng/day]** |
| --- | --- | --- | --- | --- | --- |
| **Sarego** | 42.9 | 20.5 | 5.4 | 5.6 | 11.4 |
| **Lonigo** | 51.0 | 24.2 | 4.7 | 6.2 | 15.9 |
| **Legnago** | 40.4 | 17.5 | 4.2 | 6.3 | 12.4 |
| **Veronella** | 39.1 | 18.4 | 3.4 | 5.6 | 11.7 |
| **Albaredo d’Adige** | 37.1 | 15.7 | 3.8 | 6.7 | 10.8 |
| **Tot pop** | 43.3 | 19.7 | 4.4 | 6.2 | 13.0 |
| **Men** | 42.1 | 16.0 | 4.4 | 7.4 | 14.2 |
| **Women** | 44.5 | 23.3 | 4.4 | 4.9 | 11.9 |
| **A-red area** | 48.3 | 23.0 | 4.9 | 6.0 | 14.4 |
| **B-red area** | 39.7 | 17.3 | 4.0 | 6.3 | 12.0 |

Table 16A Average total PFOA daily intake through food (A-TPI) and average PFOA intake for each food category (A-PIi) for the subjects living in each municipality, for total population (tot pop), men , women, the A-red Area and the B-red area.

| **Municipality** | **A-PTIF [ng/day]** | **A-PIF_fruit&veg_ [ng/day]** | **A-PIF_milk_ [ng/day]** | **A-PIF_meat_ [ng/day]** | **A-PIF_egg_ [ng/day]** |
| --- | --- | --- | --- | --- | --- |
| **Sarego** | 37.3 | 19.4 | 5.3 | 3.5 | 9.0 |
| **Lonigo** | 44.0 | 22.9 | 4.6 | 3.9 | 12.6 |
| **Legnago** | 34.5 | 16.6 | 4.1 | 4.0 | 9.8 |
| **Veronella** | 33.5 | 17.4 | 3.3 | 3.5 | 9.3 |
| **Albaredo d’Adige** | 31.4 | 14.8 | 3.8 | 4.2 | 8.6 |
| **Tot pop** | 37.2 | 18.7 | 4.3 | 3.9 | 10.3 |
| **Men** | 35.4 | 15.2 | 4.3 | 4.7 | 11.2 |
| **Women** | 38.9 | 22.0 | 4.3 | 3.1 | 9.5 |
| **A-red area** | 41.8 | 21.7 | 4.8 | 3.8 | 11.4 |
| **B-red area** | 33.8 | 16.4 | 3.9 | 4.0 | 9.5 |

Table 17A Average total PFOS daily intake through food (A-TPIF) and average PFOS intake for each food category (A-PIFi) for the subjects living in a municipality, for total population (tot pop), men , women, the A-red Area and the B-red area.

**PFAS total daily intake:**

PFAS total daily intake was calculated for each subject according to the following equation:

$${PTI}_{n}={PWI}_{m}+{PTIF}_{n}$$

Where:

PTI_n_ = PFAS total daily intake for the n-subject [ng/day];

PWI_m_ = PFAS intake through drinking water for the subjects living in the m-municipality [ng/day];

PTIF_n_ = PFAS total daily intake through food estimated for the n-subject [ng/day].

|  | **PFOA [ng/day]** | | | **PFOS [ng/day]** | | |
| --- | --- | --- | --- | --- | --- | --- |
| **Municipality** | **A-PTI** | **A-PTI, men** | **A-PTI, women** | **A-PTI** | **A-PTI, men** | **A-PTI, women** |
| **Sarego** | 424.6 (1930.9, AS) | 445.0 | 411.6 | 70.1 | 71.3 | 69.3 |
| **Lonigo** | 410.1 (1105.7, AS) | 449.7 | 373.0 | 71.2 | 75.5 | 67.2 |
| **Legnago** | 66.6 | 64.5 | 69.2 | 40.2 | 36.3 | 44.5 |
| **Veronella** | 137.2 | 171.6 | 119.9 | 43.1 | 43.1 | 43.1 |
| **Albaredo d’Adige** | 196.1 | 220.4 | 151.8 | 45.8 | 49.1 | 39.7 |
| **Tot pop** | 231.8 (638.1, AS) | 238.4 | 226.8 | 53.9 | 54.0 | 53.8 |
| **A-red area** | 414.9 (1377.1, AS) | 448.3 | 386.8 | 70.8 | 74.1 | 67.9 |
| **B-red area** | 98.0 | 106.2 | 89.7 | 41.5 | 39.4 | 43.5 |

Table 18A PFAS total average daily intake (A-PTI) in [ng/day] for the different municipalities and for the total population (median scenario, MS). AS = average scenario.

**Analysis of the literature**

A comprehensive review of PFAS-specific PBPK models was performed to choose several different models for the evaluation highlighting their advantages and deficits.

Moreover, the analysis of the literature was necessary to collect many values for several parameters required as input in the PBPK models in order to use them in the optimization process.

The comprehensive review of the literature (last update: 05/03/2020) was performed by searching Web of science, Scopus and PubMed. Search terms included an extensive list of perfluorinated compounds and PBPK models. Searches were performed by joining two parenthetical terms with an AND operator. The first term was composed of PFAS terms linked with OR operators, and the second with PBPK modeling terms linked with OR operators. Duplicates were removed from results.

All the PBPK models developed to simulate the ADME of PFAS in animals or humans found with this research were collected and analyzed.

| PFAS Search Terms | | Pubmed | Web of Science | Scopus |
| --- | --- | --- | --- | --- |
| Perfluorinated | Physiologically based pharmacokinetic | ((((((((((((Perfluorinated) OR (perfluorooctane sulfonate)) OR (perfluorooctanoate)) OR (Polyfluoroalkyl*)) OR (Perfluorinated*)) OR (Perfluorooctanoic acid)) OR (perfluorooctane sulfonic acid)) OR (perfluorinated acid)) OR (fluorocarbons)) OR (Perfluorinated alkyl substances)) OR (fluorinated organic compounds)) OR (PFAS)) AND (((((((Physiologically based pharmacokinetic) OR (Physiologically based toxicokinetic)) OR (Physiologically based biokinetic)) OR (PBTK)) OR (PBBK)) OR (Physiologically based kinetic)) OR (PBK)): 76 results | TOPIC:  (Perfluorinated) *OR* TOPIC:  (perfluorooctane sulfonate) *OR* TOPIC:  (perfluorooctanoate) *OR* TOPIC:  (Polyfluoroalkyl*) *OR* TOPIC:  (Perfluorinated*) *OR* TOPIC:  (Perfluorooctanoic acid) *OR* TOPIC:  (perfluorooctane sulfonic acid) *OR* TOPIC:  (perfluorinated acid) *OR* TOPIC:  (fluorocarbons) *OR* TOPIC:  (Perfluorinated alkyl substances) *OR* TOPIC:  (fluorinated organic compounds) *OR* TOPIC:  (PFAS) AND TOPIC:  (Physiologically based pharmacokinetic) *OR* TOPIC:  (Physiologically based toxicokinetic) *OR* TOPIC:  (Physiologically based biokinetic) *OR* TOPIC:  (PBTK) *OR* TOPIC:  (PBBK) *OR* TOPIC:  (PBK) *OR* TOPIC:  (Physiologically based kinetic): 35 results | (perfluorinated OR perfluorooctane sulfonate OR perfluorooctanoate OR polyfluoroalkyl* OR  perfluorinated* OR perfluorooctanoic acid OR perfluorooctane sulfonic acid OR perfluorinated acid  OR fluorocarbons OR perfluorinated alkyl substances OR fluorinated organic compounds OR pfas)  AND (physiologically based pharmacokinetic OR physiologically based toxicokinetic OR  physiologically based biokinetic OR pbtk OR pbbk OR physiologically based kinetic OR pbk):  16 results |
| perfluorooctane sulfonate | Physiologically based toxicokinetic |  |  |  |
| perfluorooctanoate | Physiologically based biokinetic |  |  |  |
| Polyfluoroalkyl* | PBTK |  |  |  |
| Perfluorinated* | PBBK |  |  |  |
| Perfluorooctanoic acid | Physiologically based kinetic |  |  |  |
| perfluorooctane sulfonic acid | PBK |  |  |  |
| perfluorinated acid |  |  |  |  |
| fluorocarbons |  |  |  |  |
| Perfluorinated alkyl substances |  |  |  |  |
| fluorinated organic compounds |  |  |  |  |
| PFAS |  |  |  |  |

**Table 19A Search terms used for comprehensive review.**

The prisma diagram is reported in the figure below:

**
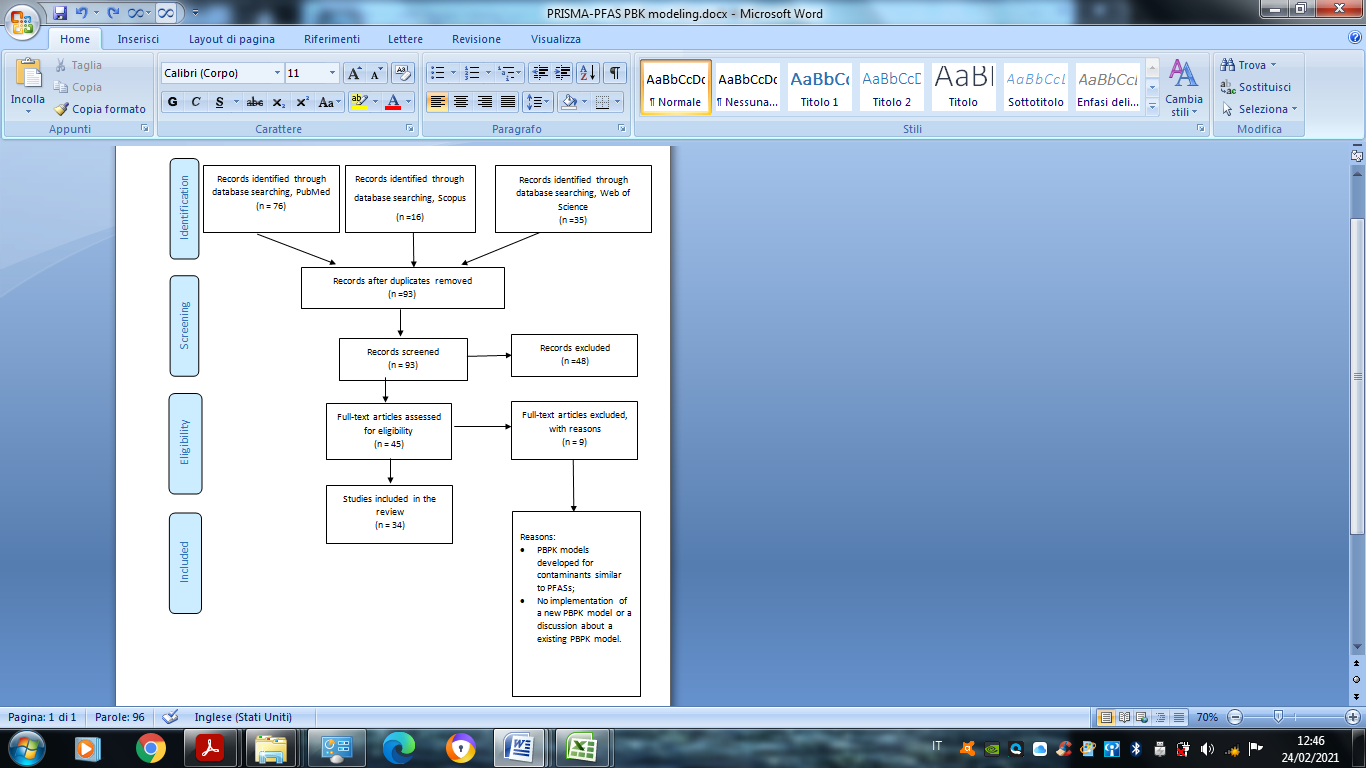
**

**Figure 1A Prisma diagram**

**model evaluation**

**parameters given as input in the “oral exposure” section of the Adapted Loccisano model:**

- the PFAS total daily intake through food estimated for the n-subject in µg/day ( = PTIFn /1000);
- the PFAS concentration in tap water in the m-municipality in µg/day ( = PCTWm /1000) for the time period P1;
- the difference between the PFAS concentration in tap water in the m-municipality in µg/day ( = PCTWm /1000) for the time period P1and the PFAS concentration in tap water in the m-municipality in µg/day ( = PCTWm /1000) for the time period P2;
- the difference between the PFAS concentration in tap water in the m-municipality in µg/day ( = PCTWm /1000) for the time period P2 and the PFAS concentration in tap water in the m-municipality in µg/day ( = PCTWm /1000) for the time period P3;
- the difference between the PFAS concentration in tap water in the m-municipality in µg/day ( = PCTWm /1000) for the time period P3and the PFAS concentration in tap water in the m-municipality in µg/day ( = PCTWm /1000) for the time period P4;
- the PFAS concentration in groundwater in the m-municipality in µg/day ( = PCGWm /1000);
- the contaminated water intake rate for the n-subject [L/day] ( = CWIRn ), that is equal to the tap water intake rate for the n-subject [L/day]; ( = TWIRn ) and equal to the groundwater intake rate for the n-subject [L/day]; ( = GWIRn ).
