# Supplementary material for: Reliability of toxicokinetic modelling for PFAS exposure assessment in contaminated water in northern Italy
Source: Heliyon. 2024 Jul 31;10(15):e35288. doi: 10.1016/j.heliyon.2024.e35288 (PMC11334853; doi:10.1016/j.heliyon.2024.e35288)
Supplement: Multimedia component 2 [file mmc2.docx]

**Annex B**

The modifications made to the original Loccisano model

First, the number of “exposure parameters” was extended, adding the time corresponding to the duration of the different exposure periods. Second, the lines of the code used to turn the dose on and off were modified to describe the beginning and the end of the exposure to PFAS concentrations in groundwater and of the different concentrations of PFAS in tap water. This operation was essential for attributing the specific PFAS concentration to the corresponding exposure period for the different scenarios. Then, in the “oral exposure” section new parameters were given as inputs in the model.

The differences between the PFAS concentration in the tap water of each municipality for a defined time period and the following one were calculated to determine the actual daily PFAS intake as input in the model.

Furthermore, in the differential equation for the gut compartment new terms were added to describe the differences in PFAS intake over time (due to the variation in the PFAS concentration in tap water). Moreover, the errors in the original code were corrected according to the suggestions of the EFSA Scientific Panel (EFSA, 2018) –i.e. PFOA half-life of 2.3 years (i.e. Tmc = 6000 μg/h/kg0.75), value of the cardiac output to liver (0.25) corrected for the cardiac output to the gut (0.069), the duration of dose from 0.6 to 24 hours and the Stiff method was used-.

The declared body weight, the predicted exposure time, the predicted PFAS oral intake, the average observed PFAS concentration in tap water and groundwater for each time period and the predicted water intake of the specific subject were used in the simulations (the average values were used in the analyses with aggregate data).

Example of the Berkeley-Madonna script for a man living in Lonigo, PFOA

METHOD Stiff

STARTTIME = 0 ; ID 11324, Lonigo

STOPTIME= 219000 ;stop simulation (h; 25 years)

DT = 0.01

; Physiological parameters (from Brown, et al)

;fractional blood flows to tissues

QCC = 12.5 ; Cardiac blood output (L/h/kg^0.75)

QFC = 0.052 ; Fraction cardiac output going to fat

QLC = 0.069 ; Fraction cardiac output going to liver

QKC = 0.175 ; Fraction cardiac output going to kidney

QfilC = 0.035 ; Fraction cardiac output to the filtrate compartment (20% of kidney blood flow)

QSkC = 0.058 ; Fraction cardiac output going to skin

QGC = 0.181 ; Fraction cardiac output going to gut

BW = 75 ; male

;fractional tissue volumes

VLC = 0.026 ; Fraction liver volume

VFC = 0.214 ; Fraction fat volume

VKC = 0.004 ; Fraction kidney volume

VfilC = 0.0004 ; Fraction filtrate compartment volume (10% of kidney volume)

VGC = 0.0171 ; Fraction gut volume

VPlasC = 0.0428 ; Fraction plasma volume

;dermal uptake

SkinTarea = 9.1*((BW*1000)**0.666) ; Total area of skin (cm^2)

Skinthickness = 0.1 ; Skin thickness (cm)

; Chemical-specific parameters (PFOA)

Tmc = 6000 ; Maximum resorption rate

Kt = 55 ; Resorption affinity; same as monkey

Free = 0.02 ; Free fraction of PFOA in plasma; same as monkey

PL = 2.2 ; Liver/plasma partition coefficient

PF = 0.04 ; Fat/plasma partition coefficient

PK = 1.05 ; Kidney/plasma partition coefficient

PSk = 0.1 ; Skin/plasma partition coefficient

PR = 0.12 ; Rest of the body/plasma partition coefficient

PG = 0.05 ;Gut/blood plasma coeff.

kurinec = 0.0003 ; Elimination rate (1/h); estimated from data of Harada, et al 2005

kurine = kurinec*BW**(-0.25)

; Free fraction of chemical in tissues

FreeL = Free/PL ;liver

FreeF = Free/PF ;fat

FreeK = Free/PK ;kidney

FreeSk = Free/PSk ;skin

FreeR = Free/PR ;rest of tissues

FreeG = Free/PG ;gut

; Exposure parameters

tchng = 58284 ; from the beginning of exposure to February 2014

T2 = 76680 ; from Feb 2014 to Apr 2016,

T3 = 89820 ; from Apr 2016 to Oct 2017, from here on the conc. in the tap water is 5 ng/L:

T4 = 91980 ; time at the sampling, 2018

;turn dose on/off

DoseOn20142016 = IF time>tchng THEN 1.0 ELSE 0.0

DoseOn20162017 = IF time>T2 THEN 1.0 ELSE 0.0

DoseOn2017 = IF time>T3 THEN 1.0 ELSE 0.0

DoseOnpozzo = IF time<T4 THEN 1.0 ELSE 0.0

;direct input to plasma (IV dose)

;IVconc = 0.0 ;iv uptake (ug/kg/day)

;IVdose = IVconc*BW ;(ug/day)

; Dermal exposure

Dermconc = 0.0 ; Dermal concentration (mg/mL)

Dermvol = 0.001 ; Dermal exposure volume (mL)

Dermdose = Dermconc*Dermvol*1000 ; (ug)

Skinarea = 972 ; Exposed area on skin (cm^2)

; Oral exposure

; Oral uptake (ug/kg/day), ho preso I valori MB (medie tra LB e UB del documento ISS)

Oraldose = 0.059 ; (ug/day), esposizione giornaliera di PFOA al cibo

Drinkconcpozzo = 1.690 ; (ug/L)

Drinkconcreteal2014 = 0.326 ; (ug/L)

Drinkconcrete20142016 = 0.108 ; difference in PFOA conc. in tap water from Feb 2014 to Apr 2016,

Drinkconcrete20162017 = 0.146 ; difference in PFOA conc. in tap water between Feb 2014-Apr 2016 ;and May 2016- Oct 2017;

Drinkconcretedal2017 = 0.067 ; difference in PFOA conc. in tap water between May 2016- Oct 2017 ;and after Oct 2017

Drinkrate = 1.5/3 ; Drinking water rate (L/day),

Drinkdosepozzo = Drinkconcpozzo*Drinkrate ; (ug/day)

Drinkdoserete2014 = Drinkconcreteal2014*Drinkrate ; (ug/day)

Drinkdoserete20142016 = Drinkconcrete20142016*Drinkrate ; (ug/day)

Drinkdoserete20162017 = Drinkconcrete20162017*Drinkrate ; (ug/day)

Drinkdoserete2017 = Drinkconcretedal2017*Drinkrate ; (ug/day)

Tinput = 24

;oral

Inputcibo = IF MOD(time,24) <=Tinput THEN Oraldose/Tinput ELSE 0.0

;drinking water

Inputpozzo = IF MOD(time,24) <= Tinput THEN Drinkdosepozzo/Tinput ELSE 0.0

Inputrete1 = IF MOD(time,24) <= Tinput THEN Drinkdoserete2014/Tinput ELSE 0.0

Inputrete2 = IF MOD(time,24) <= Tinput THEN Drinkdoserete20142016/Tinput ELSE 0.0 ; difference in PFAS intake between the time period P1 and P2

Inputrete3 = IF MOD(time,24) <= Tinput THEN Drinkdoserete20162017/Tinput ELSE 0.0; difference in PFAS intake between the time period P2 and P3

Inputrete4 = IF MOD(time,24) <= Tinput THEN Drinkdoserete2017/Tinput ELSE 0.0; difference in PFAS intake between the time period P3 and P4

; Scaling parameters

QC = QCC*BW**0.75 ;Cardiac output (L/h)

Htc = 0.44 ;hematocrit

QCP = QC*(1-Htc) ; Plasma flow

QL = QLC*QCP ; Plasma flow to liver (L/h)

QF = QFC*QCP ; Plasma flow to fat (L/h)

QK = QKC*QCP ; Plasma flow to kidney (L/h)

Qfil = 0.2*QK ; Plasma flow to filtrate compartment (L/h); 20% of QK

QG = QGC*QCP ; Plasma flow to gut (L/h)

QSk = IF Dermconc >0.0 THEN QSkC*QCP*(Skinarea/SkinTarea) else 0.0 ;plasma flow to skin

QR = QCP - QL - QF - QK - Qfil - QG -QSk ; Plasma flow to rest of the body (L/h)

Qbal = QCP - (QL+QF+QK+QFil+QG+QSk) ; balance check

VL = VLC*BW ; Liver volume (L)

VF = VFC*BW ; Fat volume (L)

VK = VKC*BW ; Kidney volume (L)

Vfil = VfilC*BW ; Fitrate compartment volume (L)

VG = VGC*BW ; Gut volume (L)

VPlas = VPlasC*BW ; Plasma volume (L)

VSk = (Skinarea*Skinthickness)/1000 ; Skin volume (L)

VR = 0.84*BW - VL - VF - VK - Vfil - VG - VPlas - VSk ; Rest of the body volume (L)

Vbal = (0.84*BW)-(VL+VF+VK+VFil+VG+Vplas+VSk) ; Balance check

Tm = Tmc*BW**0.75 ;transporter maximum

;>>>>>>>>>>>>>>Model equations<<<<<<<<<<<<<<<<<<

;Plasma compartment

APlas' = QF*CF*FreeF+(QL+QG)*CL*FreeL+QR*CR*FreeR+QSk*CSk*FreeSk+QK*CK*FreeK-QCP*CA*Free

init APlas = 0.0

CAFree = APlas/VPlas ;free concentration of chemical in plasma; ug/L (ng/mL)

CA = CAFree/Free ;total concentration of chemical in plasma

; Gut compartment

AG' = QG*(CA*Free-CG*FreeG) + Inputcibo + Inputpozzo*DoseOnpozzo + Inputrete1 - Inputrete2*DoseOn20142016 - Inputrete3*DoseOn20162017 - Inputrete4*DoseOn2017

init AG = 0.0

CG = AG/VG ; Concentration in gut (ug/L)

CVG = CG/PG ; Concentration leaving gut (ug/L)

; Liver compartment

AL' = (QL*(CA*Free))+(QG*CG*FreeG) - ((QL+QG)*CL*FreeL) ; Rate of change in liver (ug/h)

init AL = 0.0

CL = AL/VL ; Concentration in liver (ug/L)

CVL = CL/PL ; Concentration leaving liver (ug/L)

; Fat compartment

AF' = QF*(CA*Free-CF*FreeF) ; Rate of change in fat (ug/h)

init AF = 0.0

CF = AF/VF ; Concentration in fat (ug/L)

CVF = CF/PF ; Concentration leaving fat (ug/L)

; Kidney compartment

AK' = QK*(CA*Free-CK*FreeK) + (Tm*Cfil)/(Kt+Cfil) ; Rate of change in kidneys (ug/h)

init AK = 0.0

CK = AK/VK ; Concentration in kidneys (ug/L)

CVK = CK/PK ; Concentration leaving kidneys (ug/L)

; Filtrate compartment

Afil' = Qfil*(CA*Free-Cfil) - (Tm*Cfil)/(Kt+Cfil) ; Rate of change in filtrate compartment (ug/h)

init Afil = 0.0

Cfil = Afil/Vfil ; Concentration in filtrate compartment (ug/L)

; Storage compartment for urine

Adelay' = Qfil*Cfil-kurine*Adelay

init Adelay = 0.0

; Urine

Aurine' = kurine*Adelay

init Aurine = 0.0

; Skin compartment

ASk' = QSk*(CA*Free-CSk*FreeSk); Rate of change in skin(ug/h)

init ASk = 0.0

CSk = ASk/VSk ; Concentration in skin compartment (ug/L)

CVSk = CSk/PSk ; Concentration leaving skin compartment (ug/L)

; Rest of the body

AR' = QR*(CA*Free-CR*FreeR) ; Rate of change in rest of the body (ug/h)

init AR = 0.0

CR = AR/VR ; Concentration in rest of the body (ug/L)

CVR = CR/PR ; Concentration leaving rest of the body (ug/L)

Display TmC,Kt,Free,PL,PK,PF,PR,PSK,PG,tchng,BW,QCC,QFC,QLC,QKC,QGC,QSkC,VFC,VLC,VKC,VGC,VFilC,VPlasC,Drinkrate

Display CG, CL, CF,CK,CA,Cfil,CR ;for plotting
