# Supplementary material for: Reliability of toxicokinetic modelling for PFAS exposure assessment in contaminated water in northern Italy
Source: Heliyon. 2024 Jul 31;10(15):e35288. doi: 10.1016/j.heliyon.2024.e35288 (PMC11334853; doi:10.1016/j.heliyon.2024.e35288)
Supplement: Multimedia component 3 [file mmc3.docx]

## **PBTK model assessment process**

**Partition coefficients in the modified PBTK models:**

| **Model (PFOA)** | **PL** | **PF** | **PK** | **PSk** | **PR** | **PG** |
| --- | --- | --- | --- | --- | --- | --- |
| **Loccisano** | 2.2 | 0.04 | 1.05 | 0.1 | 0.12 | 0.05 |
| **ML1** | 1.03 | 0.47 | 1.17 | 0.1 | 0.12 | 0.05 |

Table 1 partition coefficients for PFOA (liver/plasma: PL, fat/plasma: PF, kidney/plasma: PK, skin/plasma: PSk, rest of the body/plasma: PR and gut/blood plasma: PG) in the original Loccisano model (Loccisano) and in Modified Loccisano model 1 (ML1).

| **Model (PFOS)** | **PL** | **PF** | **PK** | **PSk** | **PR** | **PG** |
| --- | --- | --- | --- | --- | --- | --- |
| **Loccisano** | 3.72 | 0.14 | 0.8 | 0.29 | 0.2 | 0.57 |
| **ML1** | 2.67 | 0.33 | 1.26 | 0.29 | 0.2 | 0.57 |

Table 2 partition coefficients for PFOS (liver/plasma: PL, fat/plasma: PF, kidney/plasma: PK, skin/plasma: PSk, rest of the body/plasma: PR and gut/blood plasma: PG) in the original Loccisano model (Loccisano) and in Modified Loccisano model 1 (ML1).

**Values found in literature for Kt, Tmc and Free:**

|  | **PFOA** | | | **PFOS** | | |  |
| --- | --- | --- | --- | --- | --- | --- | --- |
| **Reference** | **Kt [mg/L]** | **T_mc [mg/h/kg_^0.75^_]_** | **Free (unitless)** | **Kt [mg/L]** | **T_mc [mg/h/kg_^0.75^_]_** | **Free (unitless)** |  |
| Dzierlenga et al., 2020 | 0.055 | 4.8 | 0.02 | 0.023 | 3.27 | 0.025 |  |
| Wu et al.,  2015 | mean:0.055; lower-upper bounds: not available; distribution: not available | mean:4.8; lower-upper bounds: 1.67-11.0; distribution: Log normal | mean: 0.02; lower-upper bounds: 0.007-0.046; distribution: Log normal | mean: 0.023; lower-upper bounds: not available; distribution: not available | mean: 3.27; lower-upper bounds: 1.1-7.5; distribution: Log normal | mean: 0.025; lower-upper bounds: 0.0087-0.058; distribution: Log normal |  |
| Ruark et al., 2017 | mean:0.055; lower-upper bounds: not available; distribution: not available | mean:4.8; lower-upper bounds: 1.77-13.0; distribution: Log normal | mean: 0.02; lower-upper bounds: 0.007-0.054; distribution: Log normal | mean: 0.023; lower-upper bounds: not available; distribution: not available | mean: 3.27; lower-upper bounds: 1.2-8.9; distribution: Log normal | mean: 0.025; lower-upper bounds: 0.009-0.068; distribution: Log normal |  |
| Loccisano et al., 2011 | 0.055 | 6 (half life = 2.3 y) and 10 (half life = 3.8 y) | 0.02 | 0.023 | 3.5 | 0.025 |  |
| Loccisano, et al., 2013 | 0.055 | 10 | 0.02 | 0.023 | 3.5 | 0.025 |  |
| Verner, et al., 2015 | 0.055 | 10 | 0.02 | 0.023 | 3.5 | 0.025 |  |
| Fàbrega et al., 2014; 2015 | 0.116 [μg/L] | 147 [μg/h], 3.60 | 0.03 | 0.0176 [μg/L] | 86.0 [μg/h], 2.08 | 0.03 |  |
| Fabrega, et al., 2016 | 0.116 [min: 1.12E-4, max: 3.0E-02] [μg/L] | 6 [min: 1.46, max: 20.9] [μg/h] | 0.03 | 0.018 [min: 3.30E-7, max: 5.0E-02] [μg/L] | 3.50 [min: 0.617, max: 17.2] [μg/h] | 0.03 |  |

Table 3 values of several parameters proposed for humans found during the research of the PBTK models for PFAS in the literature . Kt = resorption affinity, Tmc = maximum resorption rate, Free = free fraction of chemical in plasma.

**Values assumed by the N parameter:**

$N=7.40\cdot{10}^{-7}\pm0.05\cdot{10}^{-7}$, for the municipality of Sarego

$N=7.90\cdot{10}^{-7}\pm0.05\cdot{10}^{-7}$, for the municipality of Lonigo

$N=8.20\cdot{10}^{-7}\pm0.05\cdot{10}^{-7}$, for the municipality of Veronella

$N=8.35\cdot{10}^{-7}\pm0.05\cdot{10}^{-7}$, for the municipality of Albaredo d’Adige

$N=8.75\cdot{10}^{-7}\pm0.05\cdot{10}^{-7}$, for the municipality of Legnago.

$N=8.2\cdot{10}^{-7}\pm0.05\cdot{10}^{-7}$, for all women (weighted average).

The precision of N_fert_ values was lower than 0.25*10^-7^ (precision of PFOS serum concentration: 0.1 µg/L).
