# Supplementary material for: Reliability of toxicokinetic modelling for PFAS exposure assessment in contaminated water in northern Italy
Source: Heliyon. 2024 Jul 31;10(15):e35288. doi: 10.1016/j.heliyon.2024.e35288 (PMC11334853; doi:10.1016/j.heliyon.2024.e35288)
Supplement: Multimedia component 4 [file mmc4.docx]

Example of the Berkeley-madonna script for the women living in Lonigo, ML29, PFOS

METHOD Stiff

STARTTIME = 0 ; ID Lonigo, women

STOPTIME=438000 ;end of simulation (h); 50 years

DT = 0.01

; Physiological parameters (from Brown, et al 1997)

;fractional blood flows

QCC = 12.5 ; Cardiac blood output (L/h/kg^0.75)

QFC = 0.052 ; Fraction cardiac output going to fat

QLC = 0.069 ; Fraction cardiac output going to liver

QKC = 0.175 ; Fraction cardiac output going to kidney

;QfilC = 0.035 ; Fraction cardiac output to the filtrate compartment (20% of kidney blood flow)

QSkC = 0.058 ; Fraction cardiac output going to skin

QGC = 0.181 ; Fraction cardiac output going to gut

BW = 60.70 ; Body weight (kg), male

;fractional tissue volumes

VLC = 0.026 ; Fraction liver volume

VFC = 0.214 ; Fraction fat volume

VKC = 0.004 ; Fraction kidney volume

VfilC = 0.0004 ; Fraction filtrate compartment volume (10% of kidney volume)

VGC = 0.0171 ; Fraction gut volume

VPlasC = 0.0428 ; Fraction plasma volume (58% of blood)

Htc = 0.44 ; hematocrit

;for dermal exposure

SkinTarea = 9.1*((BW*1000)**0.666) ; Total area of skin (cm^2)

Skinthickness = 0.1 ; Skin thickness (cm)

; Chemical-specific parameters (PFOS)

Tmc = 3270 ; Maximum resorption rate

Kt = 23.0 ; Resorption affinity

Free = 0.03 ; Free fraction of PFOS in plasma

PL = 2.67 ; Liver/blood partition coefficient

PF = 0.33 ; Fat/blood partition coefficient

PK = 1.26 ; Kidney/blood partition coefficient

PSk = 0.29 ; Skin/blood partition coefficient

PR = 0.2 ; Rest of the body/blood partition coefficient

PG = 0.57 ; Gut/blood partition coeff.

kurinec = 0.001 ; urinary elimination rate constant (/h/kg^-0.25); estimated from Harada, et al 2005

kurine = kurinec*BW**(-0.25)

; Free fraction of chemical in tissues

FreeL = Free/PL ;liver

FreeF = Free/PF ;fat

FreeK = Free/PK ;kidney

FreeSk = Free/PSk ;skin

FreeR = Free/PR ;rest of tissues

FreeG = Free/PG ;gut

; Exposure parameters

tchng = 99482 ; until February 2014;

T3 = 131018 ; from February 2014 to 2017

T4 = 131750; time at the sampling, 2018;

;turn dose on/off

DoseOnpozzo = IF time<tchng THEN 1.0 ELSE 0.0

DoseOn2017 = IF time>T3 THEN 1.0 ELSE 0.0

; Dermal exposure

Dermconc = 0.0 ; Dermal concentration (mg/mL)

Dermvol = 0.0 ; Dermal exposure volume (mL)

Dermdose = Dermconc*Dermvol*1000 ; (ug)

Skinarea = 5 ; Exposed area on skin (cm^2)

; Oral exposure

; Oral uptake (ug/kg/day), MB values (average between LB and UB. ISS, 2019)

Oraldose = 0.0427 ; (ug/day), daily exposure to PFOS from food

Drinkconcpozzo = 0.037 ; (ug/L)

Drinkconcrete= 0.012 ; (ug/L)

Drinkconcretedal2017 = 0.007 ;

Drinkrate = 1.50/3 ; Drinking water rate (L/day),

Drinkdosepozzo = Drinkconcpozzo*Drinkrate ; (ug/day)

Drinkdoserete = Drinkconcrete*Drinkrate ; (ug/day)

Drinkdoserete2017 = Drinkconcretedal2017*Drinkrate ; (ug/day)

Tinput = 24 ; duration of dose (h)

;oral

Inputcibo = IF MOD(time,24) <=Tinput THEN Oraldose/Tinput ELSE 0.0

;drinking water

Inputpozzo = IF MOD(time,24) <= Tinput THEN Drinkdosepozzo/Tinput ELSE 0.0

Inputrete1 = IF MOD(time,24) <= Tinput THEN Drinkdoserete/Tinput ELSE 0.0

Inputrete4 = IF MOD(time,24) <= Tinput THEN Drinkdoserete2017/Tinput ELSE 0.0

; Scaling parameters

QC = QCC*BW**0.75 ; Cardiac output (L/h)

QCP = QC*(1-Htc) ; adjust for plasma flow

QL = QLC*QCP ; Plasma flow to liver (L/h)

QF = QFC*QCP ; Plasma flow to fat (L/h)

QK = QKC*QCP ; Plasma flow to kidney (L/h)

Qfil = 0.2*QK ; Plasma flow to filtrate compartment (L/h); 20% of QK

QG = QGC*QCP ; Plasma flow to gut (L/h)

QSk = IF Dermconc >0.0 THEN QSkC*QCP*(Skinarea/SkinTarea) else 0.0 ;plasma flow to skin

QR = QCP - QL - QF - QK - Qfil - QG -QSk ; Plasma flow to rest of the body (L/h)

Qbal = QCP - (QL+QF+QK+QFil+QG+QSk) ; balance check--better be 0

VL = VLC*BW ; Liver volume (L)

VF = VFC*BW ; Fat volume (L)

VK = VKC*BW ; Kidney volume (L)

Vfil = VfilC*BW ; Fitrate compartment volume (L)

VG = VGC*BW ; Gut volume (L)

VPlas = VPlasC*BW ; Plasma volume (L)

VSk = (Skinarea*Skinthickness)/1000 ; Skin volume (L)

VR = 0.84*BW - VL - VF - VK - Vfil - VG - VPlas - VSk ; Rest of the body volume (L)

Vbal = (0.84*BW)-(VL+VF+VK+VFil+VG+VPlas+VSk) ; Balance check--better be 0

Tm = Tmc*BW**0.75 ;transporter maximum

;>>>>>>>>>>>>>>>>>>>> Model equations <<<<<<<<<<<<<<<<<<<<<<<<<<<<

; Plasma compartment

APlas' = QF*CF*FreeF+(QL+QG)*CL*FreeL+QR*CR*FreeR+QSk*CSk*FreeSk+QK*CK*FreeK-QCP*CA*Free-0.00000080*CA*QCP

init APlas = 2.3* VPlas

CAFree = APlas/VPlas ; free concentration of PFOS in plasma in ug/L (ng/mL)

CA = CAfree/Free ; total concentration in plasma

; Gut compartment

AG' = QG*(CA*Free-CG*FreeG) + Inputcibo + Inputpozzo*DoseOnpozzo + Inputrete1- Inputrete4*DoseOn2017

init AG = 1.3*VG

CG = AG/VG ; Concentration in gut (ug/L)

CVG = CG/PG ; Concentration leaving gut (ug/L)

; Liver compartment

AL' = (QL*(CA*Free))+(QG*CG*Freeg) - ((QL+QG)*CL*FreeL) ;Rate of change in liver (ug/h)

init AL = 6.1*VL

CL = AL/VL ; Concentration in liver (ug/L)

CVL = CL/PL ; Concentration leaving liver (ug/L)

; Fat compartment

AF' = QF*(CA*Free-CF*FreeF) ; Rate of change in fat (ug/h)

init AF = 0.8*VF

CF = AF/VF ; Concentration in fat (ug/L)

CVF = CF/PF ; Concentration leaving fat (ug/L)

; Kidney compartment

AK' = QK*(CA*Free-CK*FreeK) + Tm*Cfil/(Kt+Cfil) ; Rate of change in kidneys (ug/h)

init AK = 3.5*VK

CK = AK/VK ; Concentration in kidneys (ug/L)

CVK = CK/PK ; Concentration leaving kidneys (ug/L)

; Filtrate compartment

Afil' = Qfil*(CA*Free-Cfil) - Tm*Cfil/(Kt+Cfil) ; Rate of change in filtrate compartment (ug/h)

init Afil = 0.0

Cfil = Afil/Vfil ; Concentration in filtrate compartment (ug/L)

; Storage compartment for urine

;Adelay' = Qfil*Cfil-kurine*Adelay

;init Adelay = 0.0

; Urine

;Aurine' = kurine*Adelay

Aurine' = Qfil*Cfil - kurine*Aurine

init Aurine = 0.0

; Skin compartment

ASk' = QSk*(CA*Free-CSk*FreeSk) ; Rate of change in skin (ug/h)

init ASk = DermDose

CSk = ASk/VSk ; Concentration in skin compartment (ug/L)

CVSk = CSk/PSk ; Concentration leaving skin compartment (ug/L)

; Rest of the body

AR' = QR*(CA*Free-CR*FreeR) ; Rate of change in rest of the body (ug/h)

init AR = 0.5*VR

CR = AR/VR ; Concentration in rest of the body (ug/L)

CVR = CR/PR ; Concentration leaving rest of the body (ug/L)

Display TmC,Kt,Free,PL,PK,PF,PR,PSK,PG,tchng,BW,QCC,QFC,QLC,QKC,QGC,QSkC,VFC,VLC,VKC,VGC,VFilC,VPlasC,Drinkrate

Display CG, CL, CF,CK,CA,Cfil,CR ;for plotting
