# Supplementary material for: Reliability of toxicokinetic modelling for PFAS exposure assessment in contaminated water in northern Italy
Source: Heliyon. 2024 Jul 31;10(15):e35288. doi: 10.1016/j.heliyon.2024.e35288 (PMC11334853; doi:10.1016/j.heliyon.2024.e35288)
Supplement: Multimedia component 5 [file mmc5.docx]

### **The models derived from the Loccisano AL**

**Calculation of the average blood and plasma fluxes lost during each menstruation cycle, predicted for the women of each municipality**

First of all, the plasma volume hourly lost with menstruation was predicted as follows:

$$Q_{p,h}=N\cdot QCP$$

Where:

Q_p,h_ = average plasma volume hourly lost with menstruation (L/h), according to the predicted value of N;

N = menstruation coefficient (dimensionless);

QCP = plasma flow in the human body (L/h).

Then, the plasma flow (QCP) was rewritten as:

$$QCP=QCC\cdot(1-Htc)\cdot{BW}^{0.75}$$

Where:

QCC = cardiac blood output = 12.5 (L/(h*kg^0.75)), according to Loccisano model;

Htc = hematocrit = 0.44 (dimensionless);

BW = body weight (kg).

So, the previous equation was rewritten as follows:

$$Q_{p,h}=N\cdot QCC\cdot(1-Htc)\cdot{BW}^{0.75}$$

The plasma volume hourly lost with menstruation was multiplied by the number of hours in a day and the number of days during a menstrual cycle to obtain the plasma volume lost during a menstrual cycle:

$$Q_{p,mc}=N\cdot QCC\cdot(1-Htc)\cdot{BW}^{0.75}\cdot24\cdot29.2\cdot1000$$

Where:

Q_p,mc_ = average plasma volume lost during a menstrual cycle (mL/cycle);

24 = number of hours in one day (h/day);

29.9 = number of days per menstrual cycle (according to Verner et al., 2015 and Gomis et al., 2017 that proposed 12.5 menstruation cycles per year), (day/cycle);

1000 = number of mL in one L (mL/L);

This equation did not take into account that the simulation was run over the all time of exposure of the subjects. This fact produced an error, since the fertile age in a woman begins at 12 years of age (Verner, 2015; Gomis 2017), and so the menstrual cycles. The previous equation was so corrected adding a correction term for the fertile age, as shown here:

$$Q_{p,mc,fert}=N\cdot QCC\cdot(1-Htc)\cdot{BW}^{0.75}\cdot24\cdot29.2\cdot1000\cdot\frac{{exp}_{t}}{{age}_{fert}}$$

Where:

Q_p,mc,fert_ = average plasma volume lost with menstruation during one cycle, corrected for fertile age, (L/cycle);

24 = number of hours in one day (h/day);

29.9 = number of days per menstrual cycle (according to Verner, 2015 and Gomis, 2017; that proposed 12.5 menstruation cycles per year), (day/cycle);

1000 = number of mL in one L (mL/L);

Exp_t_ = time of exposure for women living in that municipality (years);

Age_fert_ = fertile age in women until the blood sampling (years), it was calculated as the difference between the average age of women in that municipality and the beginning of the fertile age (12, according to Verner, 2015):

$${age}_{fert}=age-12$$

The term: $\frac{{exp}_{t}}{{age}_{fert}}$ was the correction term for the fertile age. This term took into account that the value of N was found starting from simulations run over the all time of exposure and not over the period of time that begins at fertile age and ends at the time of the blood sampling.

The total volume of plasma lost in one year was obtained applying the following equation:

$$Q_{p,year,fert}=N\cdot QCC\cdot(1-Htc)\cdot{BW}^{0.75}\cdot24\cdot29.2\cdot1000\cdot\frac{{exp}_{t}}{{age}_{fert}}\cdot12.5$$

Where:

Q_p,year,fert_ = average plasma volume lost with menstruation during one year, corrected for fertile age, considering a number of 12.5 cycles per year (L/year).

The predicted values for the plasma flux were compared with those obtained in the Verner study (Verner, 2015). In this study, the value for the plasma flux lost with menstruation during one cycle was 69.4 mL, whereas the plasma flux lost with menstruation during one year was 868 mL. The corresponding average menstrual blood volume per cycle was 43.4 mL. This value was surely in an acceptable range for this parameter but it could vary a lot from women to another and to a group of women to another, mostly if the sample is small. These values for the total serum equivalent volume in menstrual fluid were obtained according to the following equation:

$$Q_{p,mc,fert}=Q_{mc,fert}\cdot0.5\cdot\left( 1-\frac{Htc}{100} \right)+Q_{mc,fert}\cdot0.5$$

Where:

Q_mc,fert_ = average menstrual fluid volume per cycle, corrected for fertility age (mL/cycle).

In the Verner study (Verner, 2015) the menstrual blood volume per cycle was assumed equal to the half of the menstrual fluid volume per cycle (Q_mc_):

$$Q_{b,mc,fert}=Q_{mc,fert}\cdot0.5$$

Where:

Q_b,mc,fert_ = average blood volume lost with menstruation during one cycle, corrected for fertile age (mL/cycle).

Thanks to this set of equations the menstrual blood volume per cycle corrected for fertility age was calculated also in this study through the following equation:

$$Q_{mc,fert}= \frac{2\cdot Q_{p,mc,fert}}{2-\frac{Htc}{100}}$$

And consequently:

$$Q_{b,mc,fert}=\frac{Q_{p,mc,fert}}{2-\frac{Htc}{100}}$$

The shared assumption in comparing the values predicted in this study using the previous set of equations and the value proposed by Verner (Verner, 2015) was that the flux of the non-blood portion of the menstrual fluid was equal to the flux of the blood portion as assumed in the Verner study (Verner, 2015). Moreover we assumed that the half of the volume that is not blood has an albumin concentration equal to that in plasma, as claimed in the Verner study (Verner, 2015). The value of hematocrit parameter (Htc) used in this study was the one proposed by Loccisano: 0.44, and not the one proposed by Verner (i.e. 40). So, the previous equation was rewritten as follows:

$$Q_{b,mc,fert}=\frac{Q_{p,mc,fert}}{2-Htc}$$

| **Municipality** | **Q_b,mc,fert_ (mL/cycle)** | **Q_p,year,fert_ (mL/year)** | **Q_p,mc,fert_ (mL/cycle)** | **Q_p,mc_ (mL/cycle)** | **Q_p,h_ (mL/h)** | **Age_fert_ (years)** | **Age (years)** | **Exp_t_ (years)** |
| --- | --- | --- | --- | --- | --- | --- | --- | --- |
| **Sarego** | 58.6 | 1143 | 91.4 | 73.9 | 1.06E-04 | 14.8 | 26.8 | 18.3 |
| **Lonigo** | 60.1 | 1173 | 93.8 | 84.3 | 1.20E-04 | 15.0 | 27.0 | 16.7 |
| **Veronella** | 78.0 | 1521 | 121.7 | 86.8 | 1.24E-04 | 14.2 | 26.2 | 19.9 |
| **Albaredo** | 96.9 | 1890 | 151.2 | 81.6 | 1.16E-04 | 9.5 | 21.5 | 17.6 |
| **Legnago** | 67.7 | 1320 | 105.6 | 96.0 | 1.37E-04 | 16.0 | 28.0 | 17.6 |
| **Women (total population)** | 67.0 | 1307 | 104.5 | 86.9 | 1.24E-04 | 14.9 | 26.9 | 17.7 |

**Table 1E Values of parameters associated to menstrual cycle calculated starting from the menstruation coefficient (N) calculated for women in each municipality. Qb,mc,fert = average blood volume lost with menstruation during one cycle, corrected for fertile age; Qp,year,fert = average plasma volume lost with menstruation during one year, corrected for fertile age, considering a number of 12.5 cycles per year; Qp,mc,fert = average plasma volume lost with menstruation during one cycle, corrected for fertile age; Qp,mc = average plasma volume lost during a menstrual cycle; Qp,h = average plasma volume hourly lost with menstruation. Agefert = fertile age in women until the blood sampling (years), it was calculated as the difference between the average age of women in that municipality and the beginning of the fertile age (12, according to Verner, 2015). Age =average age for the women living in that municipality. Expt = time of exposure for women living in that municipality.**

The following table compares the results found in this study and those reported in the Verner study:

| **Municipality** | **Q_b,mc,fert_ (mL/cycle)** | **Q_p,year,fert_ (mL/year)** | **Q_p,mc,fert_ (mL/cycle)** | **Q_p,mc_ (mL/cycle)** | **Q_p,h_ (mL/h)** |
| --- | --- | --- | --- | --- | --- |
| **This study** | 67.0 | 1307 | 104.5 | 86.9 | 1.24E-04 |
| **Verner, 2015** | 43.4 | 868 | 69.4 | - | - |

Table 2E comparison between the values found in this study and those reported in the Verner study for parameters associated with the menstrual cycle. Qb,mc,fert = average blood volume lost with menstruation during one cycle, corrected for fertile age; Qp,year,fert = average plasma volume lost with menstruation during one year, corrected for fertile age, considering a number of 12.5 cycles per year; Qp,mc,fert = average plasma volume lost with menstruation during one cycle, corrected for fertile age; Qp,mc = average plasma volume lost during a menstrual cycle; Qp,h = average plasma volume hourly lost with menstruation.

**ML2 + ML2 for women**

Of course, the relation between the different exposure times was the following:

$${exp}_{t,fert}={exp}_{t}-{exp}_{t,non-fert}$$

Where:

exp_t,fert_ = average time of exposure for women living in a certain municipality during the fertile age (years);

exp_t,non-fert_ = average time of exposure for women living in a certain municipality until the fertile age (= 12 years of age).

|  | **Concentrations in tissues in women at 12 years of age** | | | | | | **exp_t,non-fert_** |
| --- | --- | --- | --- | --- | --- | --- | --- |
| **Municipality** | **CA_12_** | **CG_12_** | **CL_12_** | **CF_12_** | **CK_12_** | **CR_12_** | **(years)** |
| **Sarego** | 4.7 | 2.7 | 12.5 | 1.5 | 7.0 | 0.9 | 3.5 |
| **Lonigo** | 2.3 | 1.3 | 6.1 | 0.8 | 3.5 | 0.5 | 1.7 |
| **Veronella** | 3.8 | 2.2 | 10.1 | 1.3 | 5.7 | 0.8 | 5.7 |
| **Albaredo** | 4.7 | 2.7 | 12.5 | 1.6 | 7.1 | 0.9 | 8.1 |
| **Legnago** | 1.4 | 0.8 | 3.7 | 0.5 | 2.1 | 0.3 | 1.6 |

Table 3E Predicted PFOS concentrations in tissues in women of 12 years of age by the ML2 model for the total population for the following compartments: plasma (CA12), gut (CG12), liver (CL12), fat (CF12), kidney (CK12), rest of the body (CR12). expt,non-fert = average time of exposure for women living in a certain municipality until the fertile age (= 12 years of age).

For example, for the plasma compartment the following equation was added:

$$init APlas = \mathrm{CA}_{12}*\mathrm{VPlas}$$

Where:

init APlas = initial value of the PFOS mass in the plasma compartment [µg];

CA12 = PFOS concentration values until 12 years of age predicted by the ML2 model for total population [µg/L];

VPlas = volume of the plasma compartment [L].

Similar equations were added in all the other compartments of the ML2 model.

**Comparison between N and N_fert_**

In the following table are reported the corrected values of N taking into account the fertile age (N_fert_) for the different municipalities:

| **Municipality** | **N_fert_** | **N** | **exp_t,fert_ (years)** | **exp_t_ (years)** |
| --- | --- | --- | --- | --- |
| **Sarego** | 7.50E-07±0.25E-07 | 7.40E-07±0.05E-07 | 14.8 | 18.3 |
| **Lonigo** | 8.00E-07±0.25E-07 | 7.90E-07±0.05E-07 | 15.0 | 16.7 |
| **Veronella** | 8.30E-07±0.25E-07 | 8.20E-07±0.05E-07 | 14.2 | 19.9 |
| **Albaredo** | 9.30E-07±0.25E-07 | 8.35E-07±0.05E-07 | 9.5 | 17.6 |
| **Legnago** | 9.10E-07±0.25E-07 | 8.75E-07±0.05E-07 | 16.0 | 17.6 |
| **Women (total population)** | 8.44E-07±0.25E-07 | 8.19E-07±0.05E-07 | 14.9 | 17.7 |

Table 4E corrected values of menstruation coefficient taking into account the fertile age (Nfert) and associated precision compared to the values of menstruation coefficient not corrected (N) and associated precision for women living in a certain municipality and for total population of women. expt,fert = average time of exposure for women living in a certain municipality during the fertile age. expt = average time of exposure for women living in a certain municipality.

|  | **N_fert,V_** | **N_fert_** | **N** | **exp_t,fert_ (years)** |
| --- | --- | --- | --- | --- |
| **Sarego** | 5.62E-07 | 7.50E-07 | 7.40E-07 | 14.8 |
| **Lonigo** | 5.84E-07 | 8.00E-07 | 7.90E-07 | 15.0 |
| **Veronella** | 4.68E-07 | 8.30E-07 | 8.20E-07 | 14.2 |
| **Albaredo** | 3.83E-07 | 9.30E-07 | 8.35E-07 | 9.5 |
| **Legnago** | 5.75E-07 | 9.10E-07 | 8.75E-07 | 16.0 |
| **Women (total population)** | 5.51E-07 | 8.44E-07 | 8.19E-07 | 14.9 |

Table 5E Comparison between the menstruation coefficient calculated starting from the average plasma concentration proposed by Verner (N_fert,V_), the menstruation coefficient taking into account fertile age (N_fert_) and the menstruation coefficient not corrected (N) for women living in a certain municipality and for the total population of women. exp_t,fert_ = average time of exposure for women living in a certain municipality during the fertile age.
