# Supplementary material for: Reliability of toxicokinetic modelling for PFAS exposure assessment in contaminated water in northern Italy
Source: Heliyon. 2024 Jul 31;10(15):e35288. doi: 10.1016/j.heliyon.2024.e35288 (PMC11334853; doi:10.1016/j.heliyon.2024.e35288)
Supplement: Multimedia component 6 [file mmc6.docx]

**Results**

Loccisano IL = Adapted Loccisano model used at individual level

Loccisano AL = Adapted Loccisano model used at aggregate level

**Individual level, Observed (HBM) vs predicted (Loccisano IL) PFOA serum concentrations:**

Figure 1 Individual level, Observed (HBM) vs predicted (combined scenario most likely scenario + average scenario (MLS+AS)) PFOA serum concentrations.

| **Municipality** |  | **Tot. Pop. [ng/mL]** | | **Men [ng/mL]** | | **Women [ng/mL]** | |
| --- | --- | --- | --- | --- | --- | --- | --- |
|  |  | **HBM** | **Loccisano IL** | **HBM** | **Loccisano IL** | **HBM** | **Loccisano IL** |
| **Sarego** | **A.V.** | 96.7 | 138.9 | 124.5 | 140.2 | 78.2 | 138.0 |
|  | **St. Dev.** | 93.5 | 59.4 | 111.8 | 73.5 | 77.7 | 50.7 |
| **Lonigo** | **A.V.** | 69.7 | 71.0 | 103.7 | 80.6 | 39.3 | 62.4 |
|  | **St. Dev.** | 54.0 | 39.7 | 53.1 | 32.3 | 32.9 | 44.1 |
| **Veronella** | **A.V.** | 71.6 | 16.4 | 129.0 | 17.1 | 42.9 | 16.0 |
|  | **St. Dev.** | 78.2 | 4.2 | 118.9 | 6.5 | 22.3 | 2.8 |
| **Albaredo** | **A.V.** | 60.8 | 23.4 | 72.2 | 24.8 | 39.8 | 20.8 |
|  | **St. Dev.** | 40.7 | 8.9 | 43.6 | 8.8 | 25.9 | 9.3 |
| **Legnago** | **A.V.** | 32.2 | 8.2 | 47.7 | 7.3 | 18.1 | 9.2 |
|  | **St. Dev.** | 42.9 | 3.3 | 54.4 | 2.9 | 24.3 | 3.4 |
| **Tot. pop.** | **A.V.** | 57.9 | 46.5 | 78.4 | 45.1 | 38.5 | 47.8 |
|  | **St. Dev.** | 62.3 | 54.9 | 71.9 | 54.7 | 44.4 | 55.7 |

Table 1 Observed (HBM) vs predicted (Loccisano IL) PFOA serum concentration: average value (A.V.) and standard deviation (St. Dev.). Combined scenario: MLS+AS. Comparison for total population (tot pop), for the group of men (men) and for the group of women (women).

Figure 2 Observed (HBM) vs predicted (Loccisano IL) PFOA serum concentration for total population (Tot. Pop.), men (M) and female (F) subjects: average. Combined scenarios: most likely scenario + average scenario (MLS+AS) and most likely scenario + median scenario (MLS+MS).

**Individual level, Observed (HBM) vs predicted (Loccisano IL) PFOA serum concentrations, median:**

|  | **Tot. Pop. [ng/mL]** | | | **Men [ng/mL]** | | | **Women [ng/mL]** | | |
| --- | --- | --- | --- | --- | --- | --- | --- | --- | --- |
| **municipality** | **HBM** | **Locc. IL (MLS+AS)** | **Locc. IL (MLS+MS)** | **HBM** | **Locc. IL (MLS+AS)** | **Locc. IL (MLS+MS)** | **HBM** | **Locc. IL (MLS+AS)** | **Locc. IL (MLS+MS)** |
| **Sarego** | 73,3 | 141 | 35,9 | 90,3 | 139,6 | 34,65 | 59,1 | 141 | 36,7 |
| **Lonigo** | 56,3 | 70,3 | 30,6 | 100,6 | 82,2 | 34,8 | 24,5 | 54,1 | 26 |
| **Veronella** | 54,6 | 15 | 15 | 111 | 15,4 | 15,4 | 41,6 | 14,8 | 14,8 |
| **Albaredo** | 63,5 | 21,1 | 21,1 | 68,1 | 24,8 | 24,8 | 42,1 | 17 | 17 |
| **Legnago** | 20,3 | 8,2 | 8,2 | 26,3 | 6,6 | 6,6 | 7,7 | 9 | 9 |
| **Total pop.** | 39,1 | 16,6 | 15,4 | 57,0 | 16,2 | 15,6 | 23,2 | 18,5 | 15,4 |

Table 2 Observed (HBM) vs predicted (Loccisano IL) PFOA serum concentration for each municipality: median. Combined scenarios: most likely scenario + average scenario (MLS+AS) and most likely scenario + median scenario (MLS+MS).

**Individual level, Observed (HBM) vs predicted (Loccisano IL) PFOA serum concentrations, deviation from the observed value, error: (%):**

| **Municipality** | **Average value** | | | **median** | | |
| --- | --- | --- | --- | --- | --- | --- |
|  | **Tot. Pop. [%]** | **Men [%]** | **Women [%]** | **Tot. Pop. [%]** | **Men [%]** | **Women [%]** |
| **Sarego** | 43.6 | 12.6 | 76.5 | 92.4 | 54.6 | 138.6 |
| **Lonigo** | 1.9 | -22.3 | 58.8 | 24.9 | -18.3 | 120.8 |
| **Veronella** | -77.1 | -86.7 | -62.7 | -72.5 | -86.1 | -64.4 |
| **Albaredo** | -61.5 | -65.7 | -47.7 | -66.8 | -63.6 | -59.6 |
| **Legnago** | -74.5 | -84.7 | -49.2 | -59.6 | -74.9 | 16.9 |

Table 3 Observed (HBM) vs predicted (Loccisano IL) PFOA serum concentration for each municipality; deviation from the observed value, error: (%).

|  | **Tot. Pop. [ng/mL]** | | **Men [ng/mL]** | | **Women [ng/mL]** | |
| --- | --- | --- | --- | --- | --- | --- |
| **Percentile** | **HBM** | **Loccisano IL** | **HBM** | **Loccisano IL** | **HBM** | **Loccisano IL** |
| **10^th^ percentile** | 6,3 | 5,8 | 15,67 | 4,84 | 3,3 | 7,3 |
| **90^th^ percentile** | 124,9 | 121,2 | 163,81 | 113,45 | 94,9 | 141 |

Table 4 Observed (HBM) vs predicted (Loccisano IL) PFOA serum concentration. Comparison of the 10th and 90th percentiles for total population (tot pop), for the group of men (men) and for the group of women (women). Scenario: MLS+AS.

| **Wilcoxon rank sum test with continuity correction** | | | |
| --- | --- | --- | --- |
|  | **W** | **p-value** | **H_0_** |
| **Men** | 3184 | 3.5E-07 | rejected |
| **Women** | 1831 | 0.30 | Not rejected |
| **A-red area** | 1079 | 4.7E-02 | rejected |
| **B-red area** | 4820 | 1.9E-12 | rejected |
| **Tot pop** | 10262 | 1.5E-03 | rejected |

Table 5 results of the Wilcoxon rank sum test with continuity correction. W = statistic, p-value, H_0_ = null hypothesis.

**Individual level, Pearson correlation indexes:**

|  | **Pearson, tot pop** | **Pearson, men** | **Pearson, women** |
| --- | --- | --- | --- |
| **Sarego** | 0.47 | 0.51 | 0.44 |
| **Lonigo** | 0.26 | 0.26 | 0.08 |
| **Veronella** | -0.32 | -0.55 | -0.12 |
| **Albaredo** | 0.04 | 0.07 | -0.48 |
| **Legnago** | 0.01 | 0.13 | 0.10 |
| **A-red area** | 0.41 | 0.43 | 0.41 |
| **B-red area** | 0.18 | 0.19 | 0.23 |
| **Tot pop** | 0.42 | 0.46 | 0.47 |

Table 6 Pearson correlation indexes (Pearson) for the municipalities in the red area, HMB vs Loccisano IL (MLS+AS) for the total population, men , women, the A-red area and the B-red area.

**observed average PFOA serum concentration vs predicted average PFOA serum concentration in the difference scenarios:**

| **PFOA serum concentration** | | | | | | |
| --- | --- | --- | --- | --- | --- | --- |
|  | **HBM (average) [ng/mL]** | **WCS+AS [ng/mL]** | **MLS+AS [ng/mL]** | **MLS+AS (%)** | **BCS+AS [ng/mL]** | **BCS+AS (%)** |
| **Sarego** | 96.7 | 316.3 | 138.9 | 43 | 11.7 | 4 |
| **Lonigo** | 69.7 | 151.6 | 71.0 | 46 | 19.4 | 13 |
| **Legnago** | 32.2 | 9.6 | 8.2 | 88 | 8.0 | 82 |

Table 7 Average observed PFOA serum concentration (HBM), average PFOA serum concentration predicted for the worst case scenario + the average scenario (WCS+AS), for the most likely scenario + the average scenario (MLS+AS) and for the best case scenario + the average scenario (BCS+AS), and the values of MLS+AS and BCS+AS respect to the WCS+AS (percentages).

|  | **HBM (average)** | **Average PFOA serum concentration (error, %)** | | |
| --- | --- | --- | --- | --- |
|  | **[ng/mL]** | **WCS+AS** | **MLS+AS** | **BCS+AS** |
| **Sarego** | 96.7 | 227 | 44 | -88 |
| **Lonigo** | 69.7 | 118 | 2 | -72 |
| **Legnago** | 32.2 | -70 | -75 | -75 |

Table 8 Deviation from the average observed PFOA serum concentration (HBM): (error,%) for average PFOA serum concentration predicted for the worst case scenario + the average scenario (WCS+AS), for the most likely scenario + the average scenario (MLS+AS) and for the best case scenario + the average scenario (BCS+AS).

Figure 3 Comparison between the PFOA serum concentration predicted in the worst case scenario (WCS+AS) and in the best-case scenario (BCS) with the observed data (HBM) for the subjects in the A-red area.


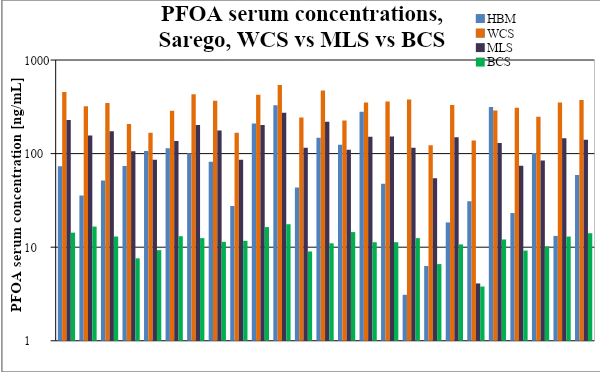


Figure 4 comparison of PFOA serum concentrations observed in the human biomonitoring study (HBM) and predicted for the combined scenarios (worst case scenario + the average scenario (WCS+AS), most likely scenario + the average scenario (MLS+AS) and best case scenario + the average scenario (BCS+AS)) for the subjects living in the municipality of Sarego. Logarithmic scale for y-axis.

###

### **PFOA concentrations in other tissues**

| **Municipality** | **CA [ng/mL]** | **CG [ng/mL]** | **CL [ng/mL]** | **CF [ng/mL]** | **CK [ng/mL]** | **CR [ng/mL]** |
| --- | --- | --- | --- | --- | --- | --- |
| **Sarego** | 138.9 | 6.9 | 305.6 | 5.6 | 175.0 | 16.7 |
| **Lonigo** | 71.0 | 3.5 | 156.2 | 2.8 | 89.4 | 8.5 |
| **Veronella** | 16.4 | 0.8 | 36.0 | 0.7 | 20.6 | 2.0 |
| **Albaredo** | 23.4 | 1.2 | 51.4 | 0.9 | 29.5 | 2.8 |
| **Legnago** | 8.2 | 0.4 | 18.1 | 0.3 | 10.3 | 1.0 |
| **Tot pop** | 46.5 | 2.3 | 102.2 | 1.9 | 58.6 | 5.6 |

Table 9 average PFOA concentrations predicted in the following compartments of the Loccisano model: plasma (CA), gut (CG), liver (CL), fat (CF), kidney (CK) and rest of the body (CR).

### **Analysis of the population subsample:**

|  | **Tot pop** | **men** | **women** |
| --- | --- | --- | --- |
| **HBM** | 7.6% | 9.6% | 1.8% |
| **Loccisano IL** | 6.7% | 7.8% | 5.6% |

Table 10 Differences in considering Average PFOA serum concentration (%) predicted with the analysis involving the total population (T. P.) and not with the analysis involving only the population subsample (P.S.), which included only the subjects with age (A) > 20 years and exposure time (E) > 10 years. Values for the Most likely scenario plus the average scenario (MLS+AS). HBM = Human bio-monitoring study. IL = Individual Level. Scenario: MLS+AS.

The Loccisano model predicted PFOA serum concentrations for the total population and for the population subsample committing approximately the same relative errors, as shown in the following table:

|  | **Tot pop** | | **men** | | **women** | |
| --- | --- | --- | --- | --- | --- | --- |
|  | **T.P.** | **P.S. (A > 20y, E > 10y)** | **T.P.** | **P.S. (A > 20y, E > 10y)** | **T.P.** | **P.S. (A > 20y, E > 10y)** |
| **error** | -19.7% | -20.4% | -42.5% | -43.4% | 24.2% | 28.8% |
| **difference** | -0.7% | | -0.9% | | 4.7% | |

Table 11 Relative errors (difference from the observed data) in predicted average PFOA serum concentrations (%) for the total population (T.P.) and for the population subsample (P.S.) and errors difference: (error associated to the simulation of the total population – error associated to the simulation of the population subsample). Scenario: MLS+AS.

**Individual level, Observed (HBM) vs predicted (Loccisano IL) PFOA serum concentrations, median, MLS+MS:**

| **municipality** | **Tot. Pop.** | | **Men** | | **Women** | |
| --- | --- | --- | --- | --- | --- | --- |
|  | **HBM** | **Loccisano IL** | **HBM** | **Loccisano IL** | **HBM** | **Loccisano IL** |
| **Sarego** | 73,3 | 35,9 | 90,3 | 34,7 | 59,1 | 36,7 |
| **Lonigo** | 56,3 | 30,6 | 100,6 | 34,8 | 24,5 | 26 |
| **Veronella** | 54,6 | 15 | 111 | 15,4 | 41,6 | 14,8 |
| **Albaredo** | 63,5 | 21,1 | 68,1 | 24,8 | 42,1 | 17 |
| **Legnago** | 20,3 | 8,2 | 26,3 | 6,6 | 7,7 | 9 |
| **Total** | 37,2 | 17,3 | 52,7 | 17,7 | 24,8 | 17,0 |

Table 12 Observed (HBM) vs predicted (Loccisano IL) PFOA serum concentration for each municipality at individual level: median. Combined scenario: most likely scenario + median scenario (MLS+MS).

**PFOA serum concentrations using the MLS+MS and the MLS+AS scenarios:**


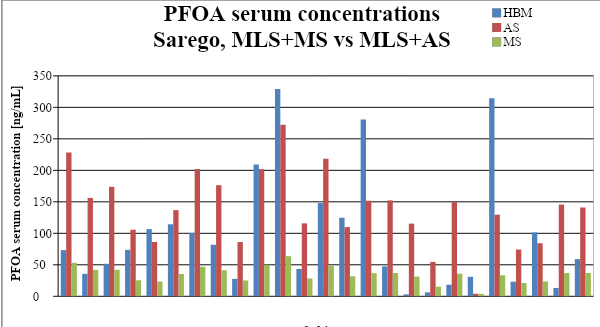


Figure 5 Comparison of PFOA serum concentrations for the subjects living in the municipality of Sarego at individual level between the observed values (HBM), the predicted values for the Most Likely Scenario + the Median Scenario (MLS+MS) and the Most Likely Scenario + the Average Scenario (MLS+AS).

## **Analysis at aggregate level**

### **PFOA**

|  | **PFOA serum concentration comparison at aggregate level [ng/mL]** | | | | | |
| --- | --- | --- | --- | --- | --- | --- |
| **Municipality** | **HBM (average)** | **ML2** | **ML1** | **Loccisano AL** | **Thompson** | **Bartell** |
| **Sarego** | 96.7 | 78.0 | 46.2 | 40.6 | 46.9 | 46.4 |
| **Lonigo** | 69.7 | 63.4 | 38.2 | 34 | 39.7 | 38.5 |
| **Veronella** | 71.6 | 27.5 | 17.5 | 17.0 | 13.8 | 12.2 |
| **Albaredo** | 60.8 | 39.3 | 24.5 | 23.2 | 20.0 | 18.9 |
| **Legnago** | 32.2 | 13.0 | 8.6 | 8.6 | 6.2 | 4.3 |
| **Tot pop** | 57.8 | 40.0 | 24.5 | 22.3 | 23.3 | 21.8 |
| **A-red area** | 78.6 | 68.2 | 40.8 | 36.2 | 42.1 | 41.1 |
| **B-red area** | 42.6 | 19.4 | 12.5 | 12.2 | 9.5 | 7.8 |

Table 13 Average PFOA concentration comparison at aggregate level [ng/mL]. HBM = Human Bio-monitoring study, ML2 = Modified Loccisano Version 2, ML1 = Modified Loccisano Version 1, Loccisano AL = Loccisano model at aggregate level, Thompson = Thompson model, Bartell = Bartell model.

| **Tot. pop.** | **Error (%)** | | | | |
| --- | --- | --- | --- | --- | --- |
| **Municipality** | **ML2** | **ML1** | **Loccisano AL** | **Thompson** | **Bartell** |
| **Sarego** | -19 | -52 | -58 | -51 | -52 |
| **Lonigo** | -9 | -45 | -51 | -43 | -45 |
| **Veronella** | -62 | -76 | -76 | -81 | -83 |
| **Albaredo** | -35 | -60 | -62 | -67 | -69 |
| **Legnago** | -60 | -73 | -73 | -81 | -87 |
| **total pop** | -38 | -61 | -64 | -65 | -68 |
| **A-red area** | -12 | -48 | -53 | -46 | -47 |
| **B-red area** | -56 | -71 | -72 | -79 | -83 |

Table 14 Average PFOA concentration comparison at aggregate level, deviation from the observed value: error (%).ML2 = Modified Loccisano Version 2, ML1 = Modified Loccisano Version 1, Loccisano AL = Loccisano model at aggregate level, Thompson = Thompson model, Bartell = Bartell model.

| **men** | **PFOA serum concentration comparison at aggregate level [ng/mL]** | | | | |
| --- | --- | --- | --- | --- | --- |
| **Municipality** | **HBM (average)** | **ML2** | **Loccisano AL** | **Thompson** | **Bartell** |
| **Sarego** | 124.5 | 74.5 | 39.4 | 43.8 | 45.5 |
| **Lonigo** | 103.7 | 62.7 | 34.2 | 38.3 | 39.1 |
| **Veronella** | 129 | 29.4 | 18.1 | 14.5 | 14.3 |
| **Albaredo** | 72.2 | 41 | 24.3 | 20.5 | 20.4 |
| **Legnago** | 47.7 | 11.4 | 7.5 | 5.4 | 4.3 |
| **Men (tot)** | 79.4 | 37.3 | 21.1 | 21.1 | 21.1 |
| **A-red area** | 109.8 | 66.2 | 35.7 | 39.9 | 41.0 |
| **B-red area** | 60.2 | 19.1 | 11.9 | 9.3 | 8.5 |

Table 15 Average PFOA concentration comparison at aggregate level for the group of men [ng/mL]. HBM = Human Bio-monitoring study, ML2 = Modified Loccisano Version 2, Loccisano AL = Loccisano model at aggregate level, Thompson = Thompson model, Bartell = Bartell model.

| **men** | **Error (%)** | | | |
| --- | --- | --- | --- | --- |
| **Municipality** | **ML2** | **Loccisano AL** | **Thompson** | **Bartell** |
| **Sarego** | -40 | -68 | -65 | -63 |
| **Lonigo** | -40 | -67 | -63 | -62 |
| **Veronella** | -77 | -86 | -89 | -89 |
| **Albaredo** | -43 | -66 | -72 | -72 |
| **Legnago** | -76 | -84 | -89 | -91 |
| **Men (tot)** | -53 | -73 | -73 | -73 |
| **A-red area** | -40 | -67 | -64 | -63 |
| **B-red area** | -68 | -80 | -85 | -86 |

Table 16 Average PFOA concentration comparison at aggregate level for the group of men; deviation from the observed value: error (%).ML2 = Modified Loccisano Version 2, ML1 = Modified Loccisano Version 1, Loccisano AL = Loccisano model at aggregate level, Thompson = Thompson model, Bartell = Bartell model.

Figure 6 Observed vs predicted PFOA serum concentration comparison at the aggregate level for the group of men [ng/mL]. HBM = Human Bio-monitoring study, ML2 = Modified Loccisano Version 2, Loccisano AL = Adapted Loccisano model used at the aggregate level, Thompson = Thompson model, Bartell = Bartell model.

| **women** | **PFOA serum concentration comparison at aggregate level [ng/mL]** | | | | | |
| --- | --- | --- | --- | --- | --- | --- |
| **Municipality** | **HBM (average)** | **ML2** | **ML18** | **Loccisano AL** | **Thompson** | **Bartell** |
| **Sarego** | 78.2 | 78.0 | 64.0 | 40.6 | 49.5 | 42.7 |
| **Lonigo** | 39.3 | 64.1 | 52.6 | 33.8 | 41.1 | 34.6 |
| **Veronella** | 42.9 | 26.4 | 21.6 | 16.3 | 13.4 | 9.9 |
| **Albaredo** | 39.8 | 36.0 | 29.5 | 21.0 | 18.8 | 15.1 |
| **Legnago** | 18.1 | 15.2 | 12.5 | 10.0 | 7.3 | 4.0 |
| **Women (tot)** | 38.5 | 42.7 | 35.0 | 23.5 | 25.7 | 20.8 |
| **A-red area** | 53.2 | 69.1 | 56.7 | 36.2 | 44.1 | 37.5 |
| **B-red area** | 25.8 | 20.0 | 16.4 | 12.6 | 10.0 | 6.6 |

Table 17 Average PFOA serum concentration comparison at aggregate level for the group of women [ng/mL]. HBM = Human Bio-monitoring study, ML2 = Modified Loccisano Version 2, ML18% = Modified Loccisano minus 18%, Loccisano AL = Loccisano model at aggregate level, Thompson = Thompson model, Bartell = Bartell model.

| **women** | **Error (%)** | | | | |
| --- | --- | --- | --- | --- | --- |
| **Municipality** | **ML2** | **ML18** | **Loccisano AL** | **Thompson** | **Bartell** |
| **Sarego** | 0 | -18 | -48 | -37 | -45 |
| **Lonigo** | 63 | 34 | -14 | 5 | -12 |
| **Veronella** | -38 | -50 | -62 | -69 | -77 |
| **Albaredo** | -10 | -26 | -47 | -53 | -62 |
| **Legnago** | -16 | -31 | -45 | -60 | -78 |
| **Women (tot)** | 11 | -9 | -39 | -33 | -46 |
| **A-red area** | 30 | 7 | -32 | -17 | -30 |
| **B-red area** | -22 | -36 | -51 | -61 | -75 |

Table 18 Average PFOA serum concentration comparison at aggregate level for the group of women; deviation from the observed value: error (%).ML2 = Modified Loccisano Version 2, ML1 = Modified Loccisano Version 1, Loccisano AL = Loccisano model at aggregate level, Thompson = Thompson model, Bartell = Bartell model.

Figure 7 Observed vs predicted PFOA serum concentration comparison at the aggregate level for the group of women [ng/mL]. HBM = Human Bio-monitoring study, ML2 = Modified Loccisano Version 2, ML18 = ML2 minus 18%, Loccisano AL = Adapted Loccisano model used at the aggregate level, Thompson = Thompson model, Bartell = Bartell model.

| **B-red area** | **PFOA serum concentration comparison at aggregate level [ng/mL]** | | | | | |
| --- | --- | --- | --- | --- | --- | --- |
|  | **HBM (average)** | **ML2** | **Loccisano AL** | **Thompson** | **Bartell** | **ML18** |
| **total pop** | 42.6 | 19.4 | 12.2 | 9.5 | 7.8 | - |
| **Men** | 60.2 | 19.1 | 11.9 | 9.3 | 8.5 | - |
| **women** | 25.8 | 20.0 | 12.6 | 10.0 | 6.6 | 16.4 |

Table 19 Average PFOA serum concentration comparison at aggregate level [ng/mL] in the B-red area for the total population, for the group of men and for the group of women. HBM = Human Bio-monitoring study, ML2 = Modified Loccisano Version 2, ML1 = Modified Loccisano Version 1, Loccisano AL = Loccisano model at aggregate level, Thompson = Thompson model, Bartell = Bartell model.

| **B-red area** | **Errors (%)** | | | | |
| --- | --- | --- | --- | --- | --- |
|  | **ML2** | **Loccisano AL** | **Thompson** | **Bartell** | **ML18** |
| **total pop** | -56 | -72 | -79 | -83 | - |
| **Men** | -68 | -80 | -85 | -86 | - |
| **women** | -22 | -36 | -51 | -61 | -75 |

Table 20 Average PFOA serum concentration comparison at aggregate level in the B-red area for the total population, for the group of men and for the group of women; deviation from the observed value: error (%). ML2 = Modified Loccisano Version 2, ML1 = Modified Loccisano Version 1, Loccisano AL = Loccisano model at aggregate level, Thompson = Thompson model, Bartell = Bartell model.

Figure 8 PFOA serum concentration comparison at aggregate level [ng/mL] for the total population, for the group of men and for the group of women. HBM = Human Bio-monitoring study, ML2 = Modified Loccisano Version 2, ML1 = Modified Loccisano Version 1, Loccisano AL = Loccisano model at aggregate level, Thompson = Thompson model, Bartell = Bartell model.

### **PFOS**

| **tot. pop.** | **average PFOS serum concentrations (ng/mL)** | | | | | |
| --- | --- | --- | --- | --- | --- | --- |
| **Municipality** | **HBM (average)** | **ML2** | **ML1** | **Loccisano AL** | **Thompson** | **Bartell** |
| **Sarego** | 5.7 | 8.9 | 11.1 | 11.2 | 15.3 | 9.3 |
| **Lonigo** | 5.3 | 7.8 | 9.6 | 9.7 | 13.6 | 8.2 |
| **Veronella** | 4.6 | 5.7 | 6.9 | 7.1 | 8.5 | 6.3 |
| **Albaredo** | 3.8 | 6.9 | 8.4 | 8.5 | 9.2 | 6.9 |
| **Legnago** | 3.4 | 5 | 6 | 6.1 | 7.3 | 5.8 |
| **total pop** | 4.4 | 6.6 | 8.0 | 8.1 | 10.5 | 7.1 |
| **A-red area** | 5.4 | 8.2 | 10.1 | 10.2 | 14.2 | 8.6 |
| **B-red area** | 3.6 | 5.4 | 6.5 | 6.6 | 7.8 | 6.1 |

Table 21 Average PFOS serum concentration comparison at aggregate level [ng/mL]. HBM = Human Bio-monitoring study, ML2 = Modified Loccisano Version 2, ML1 = Modified Loccisano Version 1, Loccisano AL = Loccisano model at aggregate level, Thompson = Thompson model, Bartell = Bartell model.

| **tot. pop.** | **Error (%)** | | | | |
| --- | --- | --- | --- | --- | --- |
| **Municipality** | **ML2** | **ML1** | **Loccisano AL** | **Thompson** | **Bartell** |
| **Sarego** | 56 | 95 | 96 | 168 | 63 |
| **Lonigo** | 47 | 81 | 83 | 157 | 55 |
| **Veronella** | 24 | 50 | 54 | 85 | 37 |
| **Albaredo** | 82 | 121 | 124 | 142 | 82 |
| **Legnago** | 47 | 76 | 79 | 115 | 71 |
| **total pop** | 50 | 82 | 85 | 134 | 63 |
| **A-red area** | 50 | 86 | 87 | 160 | 57 |
| **B-red area** | 49 | 80 | 83 | 115 | 68 |

Table 22 Average PFOS serum concentration comparison at aggregate level for the total population, for each municipality, for the subjects living in the A-red area and for the subjects living in the B-red area; deviation from the observed value: error (%). ML2 = Modified Loccisano Version 2, ML1 = Modified Loccisano Version 1, Loccisano AL = Loccisano model at aggregate level, Thompson = Thompson model, Bartell = Bartell model.

| **men** | **average PFOS serum concentrations (ng/mL)** | | | | | |
| --- | --- | --- | --- | --- | --- | --- |
| **Municipality** | **HBM (average)** | **ML2** | **ML1** | **Loccisano** | **Thompson** | **Bartell** |
| **Sarego** | 6.7 | 7.9 | 9.7 | 9.9 | 15.3 | 9.3 |
| **Lonigo** | 6.6 | 7.5 | 9.2 | 9.4 | 13.6 | 8.2 |
| **Veronella** | 4.3 | 4.9 | 5.8 | 5.9 | 8.5 | 6.5 |
| **Albaredo** | 4.2 | 6.0 | 7.4 | 7.5 | 9.2 | 7.0 |
| **Legnago** | 4.2 | 4.2 | 5.0 | 5.1 | 7.3 | 5.8 |
| **Men (tot)** | 5.1 | 5.8 | 7.0 | 7.1 | 10.2 | 7.0 |
| **A-red area** | 6.6 | 7.6 | 9.3 | 9.5 | 14.1 | 8.5 |
| **B-red area** | 4.2 | 4.6 | 5.6 | 5.7 | 7.8 | 6.1 |

Table 23 Average PFOS serum concentration comparison at aggregate level [ng/mL]. HBM = Human Bio-monitoring study, ML2 = Modified Loccisano Version 2, ML1 = Modified Loccisano Version 1, Loccisano AL = Loccisano model at aggregate level, Thompson = Thompson model, Bartell = Bartell model.

| **men** | **Error (%)** | | | | |
| --- | --- | --- | --- | --- | --- |
| **Municipality** | **ML2** | **ML1** | **Loccisano AL** | **Thompson** | **Bartell** |
| **Sarego** | 18 | 45 | 48 | 128 | 39 |
| **Lonigo** | 14 | 39 | 42 | 106 | 24 |
| **Veronella** | 14 | 35 | 37 | 98 | 51 |
| **Albaredo** | 43 | 76 | 79 | 119 | 67 |
| **Legnago** | 0 | 19 | 21 | 74 | 38 |
| **Men (tot)** | 12 | 35 | 38 | 96 | 39 |
| **A-red area** | 15 | 41 | 44 | 113 | 29 |
| **B-red area** | 10 | 32 | 34 | 85 | 45 |

Table 24 Average PFOS serum concentration comparison at aggregate level for men, for men in each municipality, for the group of men living in the A-red area and for the group of men living in the B-red area; deviation from the observed value: error (%). ML2 = Modified Loccisano Version 2, ML1 = Modified Loccisano Version 1, Loccisano AL = Loccisano model at aggregate level, Thompson = Thompson model, Bartell = Bartell model.

| **women** | **average PFOS serum concentrations (ng/mL)** | | | | | | | |
| --- | --- | --- | --- | --- | --- | --- | --- | --- |
| **Municipality** | **HBM (average)** | **ML29** | **MLV** | **ML2** | **ML1** | **Loccisano** | **Thompson** | **Bartell** |
| **Sarego** | 5.1 | 6.4 | 6.9 | 9 | 11.2 | 11.3 | 16.4 | 9.1 |
| **Lonigo** | 4.1 | 5.8 | 6.3 | 8.2 | 10.0 | 10.1 | 14.6 | 7.9 |
| **Veronella** | 4.7 | 4.5 | 5.1 | 6.3 | 7.6 | 7.7 | 9.5 | 6.1 |
| **Albaredo** | 3.1 | 4.3 | 5.1 | 6 | 7.3 | 7.4 | 9.7 | 6.5 |
| **Legnago** | 2.4 | 4.3 | 4.8 | 6.1 | 7.3 | 7.5 | 9.3 | 5.8 |
| **Women (tot)** | 3.6 | 5.1 | 5.6 | 7.2 | 8.8 | 8.9 | 12.1 | 7.0 |
| **A-red area** | 4.5 | 6.0 | 6.5 | 8.5 | 10.4 | 10.5 | 15.2 | 8.3 |
| **B-red area** | 3.0 | 4.3 | 4.9 | 6.1 | 7.4 | 7.5 | 9.4 | 5.9 |

Table 25 Average PFOS concentration comparison at aggregate level for women [ng/mL]. HBM = Human Bio-monitoring study, ML29 = Modified Loccisano 29%, MLV = Modified Loccisano-Verner, ML2 = Modified Loccisano Version 2, ML1 = Modified Loccisano Version 1, Loccisano AL = Loccisano model at aggregate level, Thompson = Thompson model, Bartell = Bartell model.

| **women** | **Errors (%)** | | | | | | |
| --- | --- | --- | --- | --- | --- | --- | --- |
| **Municipality** | **ML29** | **MLV** | **ML2** | **ML1** | **Loccisano** | **Thompson** | **Bartell** |
| **Sarego** | 25 | 35 | 76 | 120 | 122 | 222 | 78 |
| **Lonigo** | 41 | 54 | 100 | 144 | 146 | 256 | 93 |
| **Veronella** | -4 | 9 | 34 | 62 | 64 | 102 | 30 |
| **Albaredo** | 39 | 65 | 94 | 135 | 139 | 213 | 110 |
| **Legnago** | 79 | 100 | 154 | 204 | 213 | 288 | 142 |
| **total pop** | 47 | 63 | 108 | 152 | 157 | 242 | 102 |
| **A-red area** | 36 | 47 | 92 | 135 | 137 | 244 | 88 |
| **B-red area** | 57 | 77 | 122 | 167 | 173 | 241 | 115 |

Table 26 Average PFOS serum concentration comparison at aggregate level for the group of women; deviation from the observed value: error (%). ML29 = Modified Loccisano 29%, MLV = Modified Loccisano-Verner,ML2 = Modified Loccisano Version 2, ML1 = Modified Loccisano Version 1, Loccisano AL = Loccisano model at aggregate level, Thompson = Thompson model, Bartell = Bartell model.

### **Analysis of the concentration trend over time and of the contribution to the total uptake**


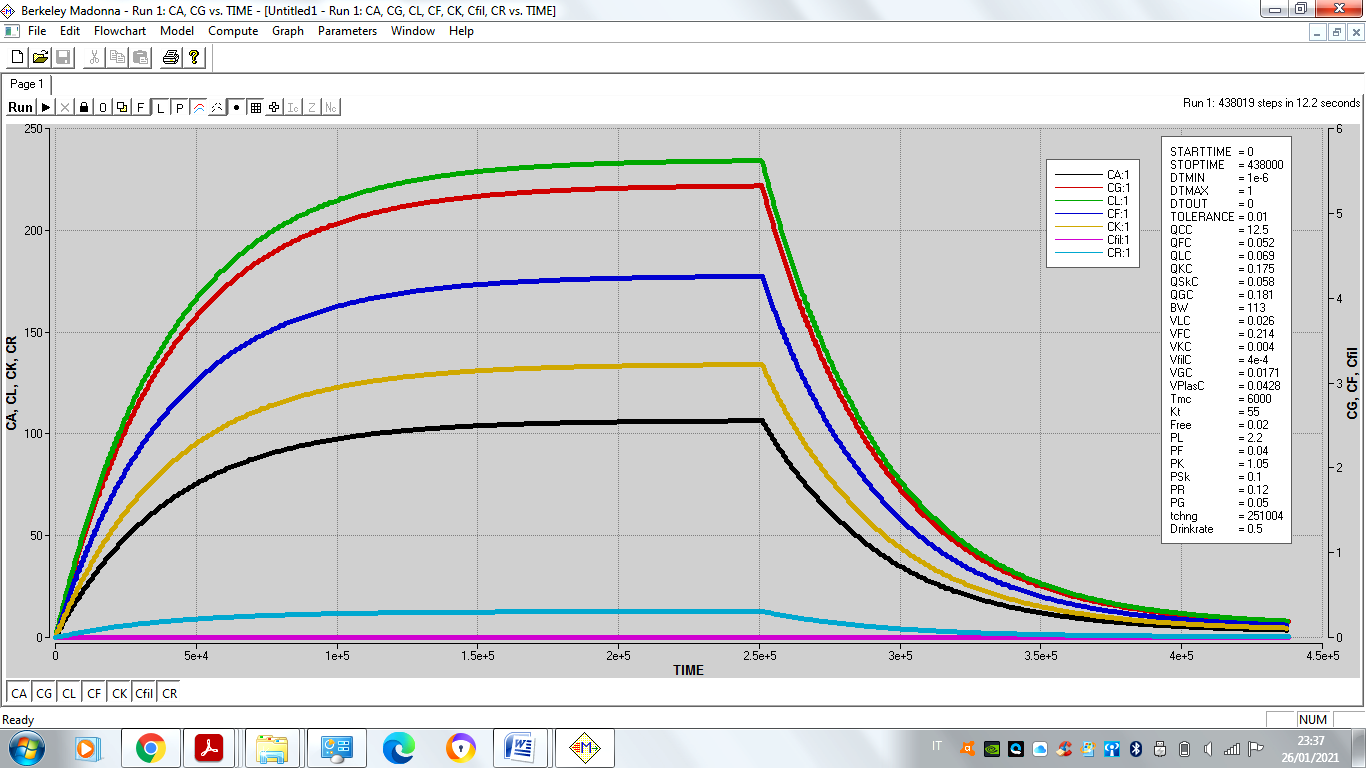


Figure 9 Loccisano model graphic output for PFOA, for the subject 24113, (man), Lonigo, MLS+AS.

|  | **Characteristics of the PFOA serum concentration curve over time respect to CAmax (%)** | | | | |
| --- | --- | --- | --- | --- | --- |
| **Municipality** | CA_bs_ | CA_ss_ | CA_Groundwater_ | CA_Tapwater_ | CA_Food_ |
| **Sarego** | 75 | 22 | 80 | 10 | 10 |
| **Lonigo** | 75 | 20 | 55 | 32 | 13 |
| **Veronella** | 96 | 50 | 0 | 73 | 27 |
| **Albaredo** | 93 | 27 | 0 | 82 | 18 |
| **Legnago** | 97 | 85 | 13 | 22 | 65 |
| **Total pop.** | 87 | 49 | 32 | 33 | 35 |
| **Men** | 88 | 51 | 30 | 34 | 37 |
| **women** | 86 | 48 | 34 | 33 | 33 |

Table 27 Percentages of the PFOA serum concentration maximum value reached over time (CAmax) for ML2, MLS+MS, total population. CAbs = PFOA serum concentration at blood sampling expressed as percentage of CAmax (%). CAss = PFOA serum concentration at steady state expressed as percentage of CAmax (%). CAGroundwater = contribution to the PFOA serum concentration maximum value derived from the exposure to groundwater expressed as percentage of CAmax (%). CATapwater = contribution to the PFOA serum concentration maximum value derived from the exposure to tap water expressed as percentage of CAmax (%). CAFood = contribution to the PFOA serum concentration maximum value derived from the exposure to food expressed as percentage of CAmax (%).

|  | **PFOS serum concentration trend over time. Concentrations respect to CA_max_ (%)** | | | | |
| --- | --- | --- | --- | --- | --- |
| **Municipality** | **CA_bs_** | **CA_ss_** | **CA_Groundwater_** | **CA_Tapwater_** | **CA_Food_** |
| **Sarego** | 88 | 64 | 22 | 28 | 50 |
| **Lonigo** | 93 | 76 | 28 | 10 | 62 |
| **Veronella** | 95 | 90 | 6 | 16 | 78 |
| **Albaredo** | 95 | 90 | 6 | 22 | 72 |
| **Legnago** | 98 | 95 | 6 | 6 | 88 |
| **Total pop.** | 95 | 84 | 15 | 13 | 73 |
| **Men** | 95 | 85 | 14 | 12 | 74 |
| **women** | 94 | 83 | 15 | 13 | 72 |

Table 28 Percentages of the PFOS serum concentration maximum value reached over time (CAmax) for ML2, MLS+MS, total population. CAbs = PFOS serum concentration at blood sampling expressed as percentage of CAmax (%). CAss = PFOS serum concentration at steady state expressed as percentage of CAmax (%). CAGroundwater = contribution to the PFOS serum concentration maximum value derived from the exposure to groundwater expressed as percentage of CAmax (%). CATapwater = contribution to the PFOS serum concentration maximum value derived from the exposure to tap water expressed as percentage of CAmax (%). CAFood = contribution to the PFOS serum concentration maximum value derived from the exposure to food expressed as percentage of CAmax (%).

## **Loccisano IL vs Loccisano AL**

Figure 10 Comparison between the average PFOA serum concentrations observed (HBM) and predicted with the Loccisano model at individual level (Loccisano IL) and at aggregate level (Loccisano AL).

Figure 11 Comparison between the median of the observed PFOA serum concentrations (HBM) and the medians predicted using the Adapted Loccisano model used at the individual level (Loccisano IL) and at the aggregate level (Loccisano AL) when adopting the MLS+MS scenario.

| **Tot pop** | **Errors in predicted PFOA average concentrations (%)** | | | | | | |
| --- | --- | --- | --- | --- | --- | --- | --- |
|  | **Sarego** | **Lonigo** | **Veronella** | **Albaredo** | **Legnago** | **Tot pop** | **B-red area** |
| **Loccisano IL, MLS+AS** | 43.6 | 1.9 | -74.5 | -61.5 | -77.2 | -19.8 | -72.1 |
| **Loccisano AL, MLS+AS** | 72.5 | 10.9 | -73.3 | -61.8 | -76.3 | -9.6 | -71.3 |

Table 29 Deviation from the observed value: errors (%) in average PFOA serum concentration predicted with Loccisano model at individual level (Loccisano IL) and at aggregate level (Loccisano AL) respect to the observed serum PFOA average concentration (HBM).

| **Men** | **Errors in predicted PFOA average concentrations (%)** | | | | | | |
| --- | --- | --- | --- | --- | --- | --- | --- |
|  | **Sarego** | **Lonigo** | **Veronella** | **Albaredo** | **Legnago** | **Tot pop** | **B-red area** |
| **Loccisano IL, MLS+AS** | 12.6 | -22.3 | -86.7 | -65.7 | -84.6 | -43.4 | -80.5 |
| **Loccisano AL, MLS+AS** | 31.5 | -24.2 | -86.0 | -66.3 | -84.3 | -40.7 | -80.3 |

Table 30 Deviation from the observed value: errors (%) in average PFOA serum concentration predicted with Loccisano model at individual level (Loccisano IL) and at aggregate level (Loccisano AL) with respect to the observed average PFOA serum concentration (HBM).

| **Women** | **Errors in predicted PFOA average concentrations (%)** | | | | | | |
| --- | --- | --- | --- | --- | --- | --- | --- |
|  | **Sarego** | **Lonigo** | **Veronella** | **Albaredo** | **Legnago** | **Tot pop** | **B-red area** |
| **Loccisano IL, MLS+AS** | 76.5 | 58.7 | -62.7 | -47.8 | -49.1 | 24.1 | -53.5 |
| **Loccisano AL, MLS+AS** | 113.3 | 92.7 | -62.0 | -47.3 | -44.9 | 47.6 | -51.1 |

Table 31 Deviation from the observed value: errors (%) in average PFOA serum concentration predicted with Loccisano model at individual level (Loccisano IL) and at aggregate level (Loccisano AL) with respect to the observed average PFOA serum concentration (HBM).

## **Parameter optimization in the ML2 and in the MLV models**

| **PFOA, women** | **parameter optimization** | | | |  |
| --- | --- | --- | --- | --- | --- |
|  | **Tmc and Kt** | | Tmc only | Kt only | **Number of women** |
| **municipality** | **Tmc (μg/h/kg^0.75^)** | **Ktc (μg/L)** | Tmc (μg/h/kg^0.75^) | Kt (μg/L) |  |
| Legnago | 12050 | 50,5 | 13100 | 42 | 33 |
| Veronella | 16800 | 39 | 23600 | 23 | 10 |
| Albaredo | 10700 | 51,5 | 11400 | 48,5 | 6 |
| **W.A.** | **12854** | **48** | 15035 | 39 |  |

Table 32 Parameter optimization (with the HBM average), PFOA, women, B-Red Area. Optimization of Maximum resorption rate and Resorption affinity constant (Tmc and Kt), optimization of Maximum resorption rate only (Tmc only) and optimization of Resorption affinity constant only (Kt only). W.A. = Weighted Average.

| **PFOS, men** | **optimized parameters** | | | |  |
| --- | --- | --- | --- | --- | --- |
|  | **Tmc and Kt** | | Tmc only | Kt only | **Number of men** |
| **municipality** | **Tmc (μg/h/kg^0.75^)** | **Ktc (μg/L)** | Tmc (μg/h/kg^0.75^) | Kt (μg/L) |  |
| Sarego | 3000 | 25,1 | 2750 | 27,1 | 10 |
| Lonigo | 3035 | 25 | 2800 | 26,7 | 24 |
| Legnago | 3270 | 23 | 3270 | 23 | 38 |
| Veronella | 3035 | 25 | 2800 | 26,7 | 5 |
| Albaredo | 2735 | 28,5 | 2200 | 34 | 11 |
| **W.A.** | **3095** | **25** | 2922 | 26 |  |

Table 33 Parameter optimization (with the HBM average), PFOS, men, B-Red Area. Optimization of Maximum resorption rate and Resorption affinity constant (Tmc and Kt), optimization of Maximum resorption rate only (Tmc only) and optimization of Resorption affinity constant only (Kt only). W.A. = Weighted Average.

| **PFOS, women** | **optimized parameters** | | | |  |
| --- | --- | --- | --- | --- | --- |
|  | **Tmc and Kt** | | Tmc only | Kt only |  |
| **municipality** | **Tmc (μg/h/kg^0.75^)** | **Ktc (μg/L)** | Tmc (μg/h/kg^0.75^) | Kt (μg/L) | **Number of women** |
| Sarego | 2785 | 27,7 | 2300 | 32,5 | 15 |
| Lonigo | 2610 | 31 | 1950 | 38,5 | 27 |
| Legnago | 2310 | 39 | 1350 | 55,5 | 33 |
| Veronella | 3085 | 24,5 | 2900 | 26 | 10 |
| Albaredo | 2535 | 32 | 1800 | 41 | 6 |
| **W.A.** | **2577** | **33** | 1885 | 42 |  |

Table 34 Parameter optimization (with the HBM average), PFOS, women. Optimization of Maximum resorption rate and Resorption affinity constant (Tmc and Kt), optimization of Maximum resorption rate only (Tmc only) and optimization of Resorption affinity constant only (Kt only). W.A. = Weighted Average.

|  | Tmc (μg/h/kg^0.75^) | Kt (μg/L) |
| --- | --- | --- |
| PFOA, women, B-red area (ML2 model) | 0,61% | -0,88% |
| PFOS, men (ML2 model) | 0,88% | -0,74% |
| PFOS, women (MLV model) | 2,19% | -1,92% |

Table 35 Influence of Kt and Tmc on the final results. The variation in the concentration estimated by the ML2 model for PFOA in women and PFOS in men, and by the MLV model for PFOS in women, was calculated with a 1% increase in the chosen parameter.

**Parameter optimization in the Thompson model**

| **PFOA** | CP from HBM [ng/mL] | **optimized kelim [day−1]** |  |
| --- | --- | --- | --- |
| municipality |  |  | N |
| Sarego | 96,7 | 0,00176 | 25 |
| Lonigo | 69,7 | 0,00123 | 51 |
| Legnago | 32,2 | 0,00015 | 71 |
| Veronella | 71,6 | 0,00015 | 15 |
| Albaredo | 60,8 | 0,00026 | 17 |
| W.A. |  | 0,00070 |  |
| W.A. corrected with median: |  | **0,00033** |  |
| **PFOS** | CP from HBM [ng/mL] | **optimized kelim [day−1]** |  |
| municipality |  |  | N |
| Sarego | 5,7 | 0,00080 | 25 |
| Lonigo | 5,3 | 0,00077 | 51 |
| Legnago | 3,4 | 0,00065 | 71 |
| Veronella | 4,6 | 0,00056 | 15 |
| Albaredo | 3,8 | 0,00072 | 17 |
| W.A. |  | **0,00070** |  |
| **MEN** | | | |
| **PFOA** |  |  |  |
|  | CP from HBM [ng/mL] | **optimized kelim [day−1]** |  |
| municipality |  |  | N |
| Sarego | 124,5 | 0,00129 | 10 |
| Lonigo | 103,7 | 0,00080 | 24 |
| Legnago | 47,7 | 0,00009 | 38 |
| Veronella | 129 | 0,00009 | 5 |
| Albaredo | 72,2 | 0,00023 | 11 |
| W.A. |  | **0,00044** |  |
| **MEN** | | | |
| **PFOS** |  |  |  |
|  | CP from HBM [ng/mL] | optimized kelim [day−1] |  |
| municipality |  |  | N |
| Sarego | 6,7 | 0,00062 | 10 |
| Lonigo | 6,6 | 0,00058 | 24 |
| Legnago | 4,2 | 0,00043 | 38 |
| Veronella | 4,3 | 0,00050 | 5 |
| Albaredo | 4,2 | 0,00064 | 11 |
| W.A. |  | **0,00052** |  |
| **WOMEN** | | | |
| **PFOA** |  |  |  |
|  | CP from HBM [ng/mL] | **optimized kelim [day−1]** |  |
| municipality |  |  | N |
| Sarego | 78,2 | 0,00230 | 15 |
| Lonigo | 39,3 | 0,00225 | 27 |
| Legnago | 18,1 | 0,00032 | 33 |
| Veronella | 42,9 | 0,00025 | 10 |
| Albaredo | 39,8 | 0,00038 | 6 |
| W.A. |  | **0,00122** |  |
| **WOMEN** | | | |
| **PFOS** |  |  |  |
|  | CP from HBM [ng/mL] | **optimized kelim [day−1]** |  |
| municipality |  |  | N |
| Sarego | 5,1 | 0,00097 | 15 |
| Lonigo | 4,1 | 0,00107 | 27 |
| Legnago | 2,4 | 0,00117 | 33 |
| Veronella | 4,7 | 0,00060 | 10 |
| Albaredo | 3,1 | 0,00094 | 6 |
| W.A. |  | **0,00103** |  |

Table 36 Optimization of the k-elimination parameter of the Thompson model, for PFOA and PFOS, obtained from the concentrations in plasma (CP) observed in the HBM study for the total population, men and women. N = number of people. W.A. = weighted average
